# Modeling the relationship of epigenetic modifications to transcription factor binding

**DOI:** 10.1093/nar/gkv255

**Published:** 2015-03-27

**Authors:** Liang Liu, Guangxu Jin, Xiaobo Zhou

**Affiliations:** Center for Bioinformatics and Systems Biology, Department of Radiology, Wake Forest School of Medicine, Winston-Salem, NC 27157, USA

## Abstract

Transcription factors (TFs) and epigenetic modifications play crucial roles in the regulation of gene expression, and correlations between the two types of factors have been discovered. However, methods for quantitatively studying the correlations remain limited. Here, we present a computational approach to systematically investigating how epigenetic changes in chromatin architectures or DNA sequences relate to TF binding. We implemented statistical analyses to illustrate that epigenetic modifications are predictive of TF binding affinities, without the need of sequence information. Intriguingly, by considering genome locations relative to transcription start sites (TSSs) or enhancer midpoints, our analyses show that different locations display various relationship patterns. For instance, H3K4me3, H3k9ac and H3k27ac contribute more in the regions near TSSs, whereas H3K4me1 and H3k79me2 dominate in the regions far from TSSs. DNA methylation plays relatively important roles when close to TSSs than in other regions. In addition, the results show that epigenetic modification models for the predictions of TF binding affinities are cell line-specific. Taken together, our study elucidates highly coordinated, but location- and cell type-specific relationships between epigenetic modifications and binding affinities of TFs.

## INTRODUCTION

Transcription factors (TFs) regulate gene expression through changes of their binding affinities to specific genomic *cis*-regulatory sequences. Analyses on TF binding sites (TFBSs) motivated the development of sequence-specific Position Weighted Matrix (PWM) approach for TFBS identification by summarizing all binding sites in the genome into 4- to 30-base-pair (bp) binding motifs, such as TRANSFAC (1) and JASPAR (2). This method enables the study of factor-specific TFBSs and sequence-specific changes of TF binding; however, it missed other related factors, such as chemical modifications to genome sequences and nearby histones (3).

Epigenetic modifications, including post-translational covalent histone modifications and DNA methylation, can mediate epigenetic regulation of gene expression, cell growth and disease development (4–9). Patterns of epigenetic modifications can serve as markers to represent gene activities and expressions, and epigenetic modifications occurring at different genome locations lead to distinct regulatory roles. Methylation of CpGs in gene promoters is generally associated with silencing of downstream genes (10–12), in contrast to that of CpGs in gene bodies. Enrichments of histone modifications, H3K4me2, H3K4me3 and H3ac, at transcription start sites (TSSs) are positively related to the extents of gene activities (4,13,14). Active *cis*-regulatory elements are marked by H3K27ac, distinguishing from inactive counterparts (15). Theoretical analysis also proved that downstream histone modifications lead to more accurate prediction of gene expression (16). To investigate the regulatory roles of histone modifications in gene expression, Chen and Gerstein (16) and other researchers (17) are the pioneers to consider location information, by dividing genome sequence into bins (16).

Epigenetic modifications have the ability to regulate gene expression, and have strong correlations with TF binding (3,18–20). Studies of associations between epigenetic modifications and TF binding showed that certain histone modifications in chromatin act on both TF access (21,22) and transcriptional initiation (23–25). For example, methylation of histones can change the activation status of DNA and thereby allow or block TFs to access the DNA (26). DNA methylation is also related to TF binding and gene silencing (11,27–30). Moreover, the usage of regulatory elements to associate TFs with DNA sequence exhibits a strong cell type-specific property (31), which is frequently related to one or more chromatin alterations (29,32–36).

Advances in development and improvement of high-throughput experimental techniques have led to enormous explosion of genomic and epigenetic data. For instance, the ENCODE project (15) generated data for >120 TFs and various types of epigenetic modifications in a number of cell lines, by using different experimental platforms. These benefited our understanding of overall changes of chromatin features around TFBSs (37–42), resulting in epigenetic modification-involved, but still sequence-specific, TF binding motifs (or PWM) for TFBS identification (31,43,44). This approach, unfortunately, failed to consider the quantitative relationships between epigenetic modifications and TF binding affinities.

**Figure 1.**
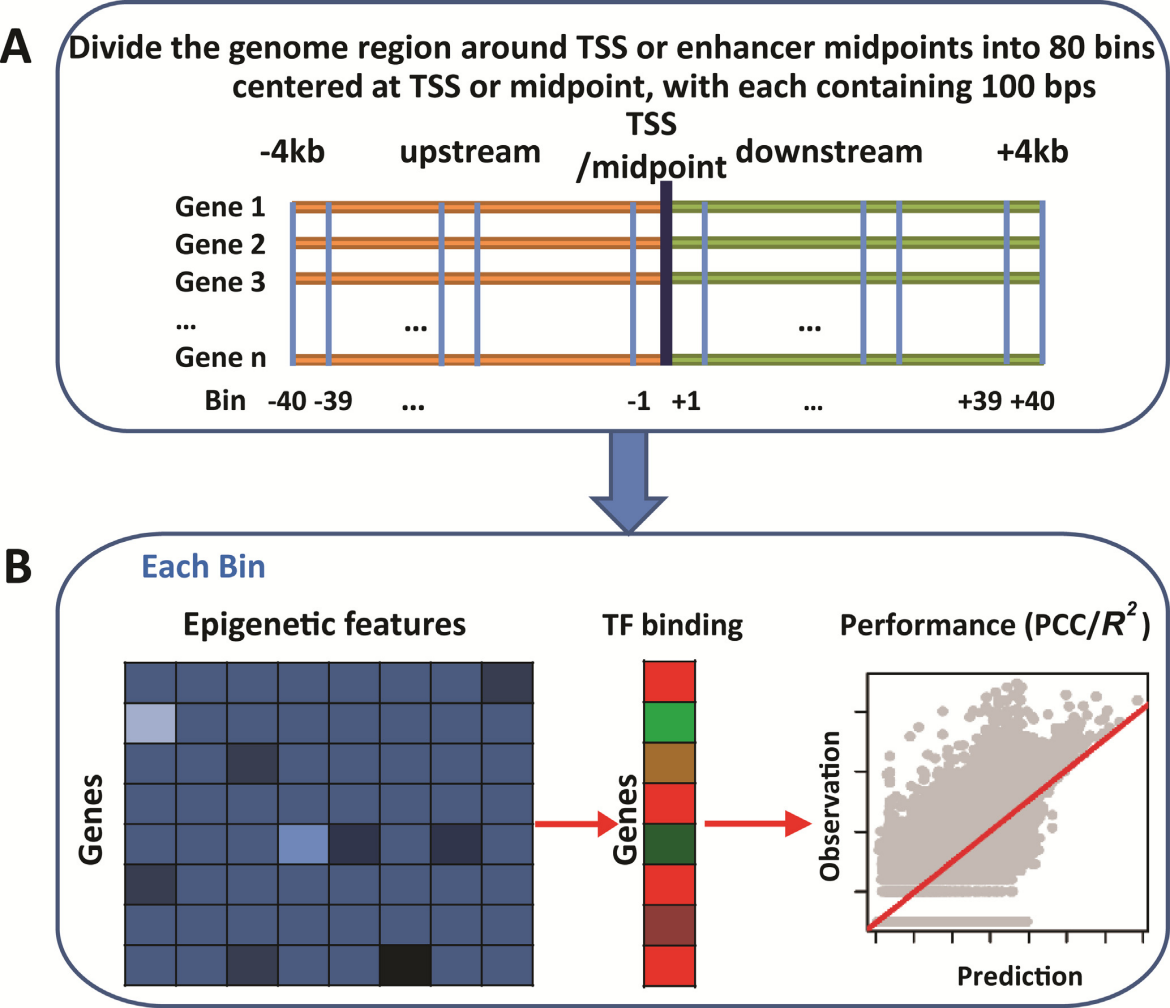
Framework of epigenetic modification model for predicting TF binding. (**A**) The 8k-bp genome regions were evenly divided into 80 bins centered at TSSs or enhancer midpoints. TF binding affinities and epigenetic modification levels were calculated in each bin based on ChIP-Seq data. (**B**) In each bin, epigenetic modification levels were used to predict TF binding affinities with the MLR and RF models. The predicted results were compared with observed values, and PCC/*R*^2^ values were used to evaluate the prediction performance.

In this paper, we present a computational approach to studying the correlations between epigenetic modifications and TF binding affinities, by taking advantage of the wealth of data from the ENCODE project (15). Instead of focusing on sequence-specific TF binding site or motif analyses, we explored quantitative relationships between epigenetic modification levels and TF binding affinities. In order to study the correlations in a combinatorial fashion, and illustrate the possible differences, we divided genome regions around TSSs (or enhancer midpoints) into bins of 100 bps to enable a location-specific study (Figure 1A). In each bin, we applied regression models to investigate the ability of epigenetic features to predict TF binding affinities. After a large-scale computational experiment, we found that, even without consideration of genome sequence, epigenetic modifications are sufficient to model TF binding affinities. Prediction accuracies vary according to genomic locations. Moreover, the contributions of epigenetic features change according to genome locations, indicating varieties exist in the relationships between epigenetic modifications and TF binding. For instance, H3K4me3, H3k9ac and H3k27ac are likely to be crucial in the regions closer to TSSs but not far from TSSs, in contrast to H3K4me1 and H3k79me2. Our analyses additionally showed that epigenetic modification models for predicting TF binding affinities are cell line-specific, indicating correlations between the two factors may vary in terms of cell conditions.

## MATERIALS AND METHODS

### Datasets

All data used in this work were downloaded from the ENCODE project (http://genome.ucsc.edu/ENCODE/downloads.html) (15). Genome-wide profiles of histone modifications, including H3K9ac, H3K27ac, H3K4me3, H3K4me2, H3K4me1, H3k79me2, H3K9me3, H3K27me3, H3K36me3 and H4K20me1, and the histone variant, H2az, were generated using the ChIP-Seq technique (45). The ENCODE project has profiled chromatin features across a few normal and immortal cell lines; however, more complete datasets are available from the K562 (erythrocytic leukemia cells), GM12878 (B-lymphoblastoid cell), H1-hESC (embryonic stem cells) and HepG2 (hepatocellular carcinoma cells) cell lines. These four cell lines and the corresponding data were selected in our analysis.

Genome-wide TF binding data were profiled by using the ChIP-Seq technique. The database includes >120 TFs in a number of cell lines. More complete data are available from the K562, GM12878, H1-hESC and HepG2 cell lines, containing 75, 69, 42 and 41 TFs, respectively (Supplemental Table S1). These TFs can be further categorized into general and sequence-specific groups. The former act cooperatively with RNA polymerase II and are involved in the transcription of a large fraction of genes (46), and the latter are bound to specific subsets of target genes (47). Our analyses included both types of TFs.

DNA methylation levels were quantitatively profiled with the RRBS technique and Infinium HumanMethylation450 BeadChip array. The former covers >1M CpG sites, while the latter measures the methylation levels for 485 577 CpG sites. DNA methylation data are available for almost all cell lines. In this work, the methylation level of each CpG is determined as the average of RRBS replicated experiments or HumanMethylation450 BeadChip data, and ∼1.3M CpGs were included.

All of these data were annotated with the human genome version hg19. Genomic locations of 47 321 protein-coding genes and 10 214 non-protein-coding genes with all information, including TSSs and transcription termination sites (TTSs), were obtained from the RefSeq database (downloaded from UCSC Genome Browser at http://genome.ucsc.edu/). We excluded those genes with sequence lengths (from TSS to TTS) less than 4k bps to ensure each gene has a sufficient downstream region, and 33 292 genes were finally selected.

The FANTOM5 project (48) provides information of 49 199 enhancers (http://fantom.gsc.riken.jp/5/). These enhancers were detected by bidirectional capped transcription, using the FANTOM5 CAGE expression atlas in 135 primary cell and 432 tissue samples from human.

### Genome-region separation around TSSs and enhancer midpoints

To understand direct correlations between epigenetic modifications and TF binding, and the influence from relative genome locations, we divided the 8k-bp genome regions around TSSs (−4 to +4 kb) into 80 bins. This approach resulted in each bin of 100 bps in size, 40 upstream (‘−’) and 40 downstream (‘+’) bins centered at TSS of each RefSeq gene with length greater than 4k bps (Figure 1). Similarly, we selected the genome regions by extending 8k bps from gene enhancer midpoints to investigate the correlations and possible changes. These 8k-bp regions were also divided into 80 bins, with each consisting of 100 bps (Figure 1).

Based on ChIP-Seq data for a TF or histone mark, we calculated the coverage of each nucleotide as number of reads covering this nucleotide. To calculate TF binding affinity and histone modification level in each bin, we averaged the coverages of the 100 nucleotides (12,16,17). Then the coverage of each bin was further normalized by computing the value of reads per million (RPM), and averages were taken from experimental replicates. For TF binding affinity, a log_2_ transformation (log_2_(RPM + 1)) was applied. If there were not ChIP-Seq reads mapped into one bin, a pseudo-count (−1) was assigned as the binding affinity instead of 0 to distinguish them from other non-zero but low-coverage bins.

For DNA methylation, we selected the methylation level of CpG site(s) mapped into the bin to compute methylation level for this bin. For bins with more than one CpG site, the average of methylation levels over these mapped CpG sites was selected to represent this bin's methylation level.

### Predicting TF binding affinity

We constructed prediction models to quantitatively investigate the relationships between epigenetic modifications and TF binding. Because we were also interested in the relative contributions of DNA methylation or other types of histone modifications to TF binding, we selected the Multiple Linear Regression (MLR) (with ‘mlr’ R package) and Random Forest (RF) models (with ‘randomForest’ R package) to construct the epigenetic modification model for predicting TF binding affinity in each bin.

The RefSeq genes were separated into a training dataset and a testing dataset. Specifically, we randomly selected two-thirds of genes ( = 22 194) as the training dataset and used the remaining one-third of genes as the testing dataset. In each bin, the MLR and RF models were built for each TF in training dataset with epigenetic modification levels as inputs and TF binding affinities as outputs, and subsequently applied to the testing dataset with the corresponding epigenetic modification levels as inputs to predict binding affinities of the same TF. We then calculated the Pearson correlation coefficient (PCC) between predicted TF binding affinities and experimental measurements. The coefficient of determination (*R*^2^) was employed as well to present prediction accuracy, representing the proportion of genes whose TF binding affinities could be explained by the model.

Cross-validation was used to estimate the prediction accuracy. The above procedure was repeated 50 times, and then the average PCC and *R*^2^ values between predicted and experimentally measured TF binding affinities were calculated to represent the predictive accuracy of epigenetic modification model in each bin.

In the RF model, we used the ‘%IncMSE’ obtained from ‘randomForest’ R package to represent the relative importance of histone modifications and DNA methylation to predict TF binding affinities. An epigenetic modification with higher ‘%IncMSE’ value contributes more in prediction. Due to the non-sense of ‘%IncMSE’ values when considering out of the current bin, we ranked epigenetic modification features by converting their ‘%IncMSE’ values to orders of relative importance within each bin. This method enables cross-bin comparisons.

## RESULTS

### Epigenetic modifications are predictive to TF binding affinities

**Figure 2.**
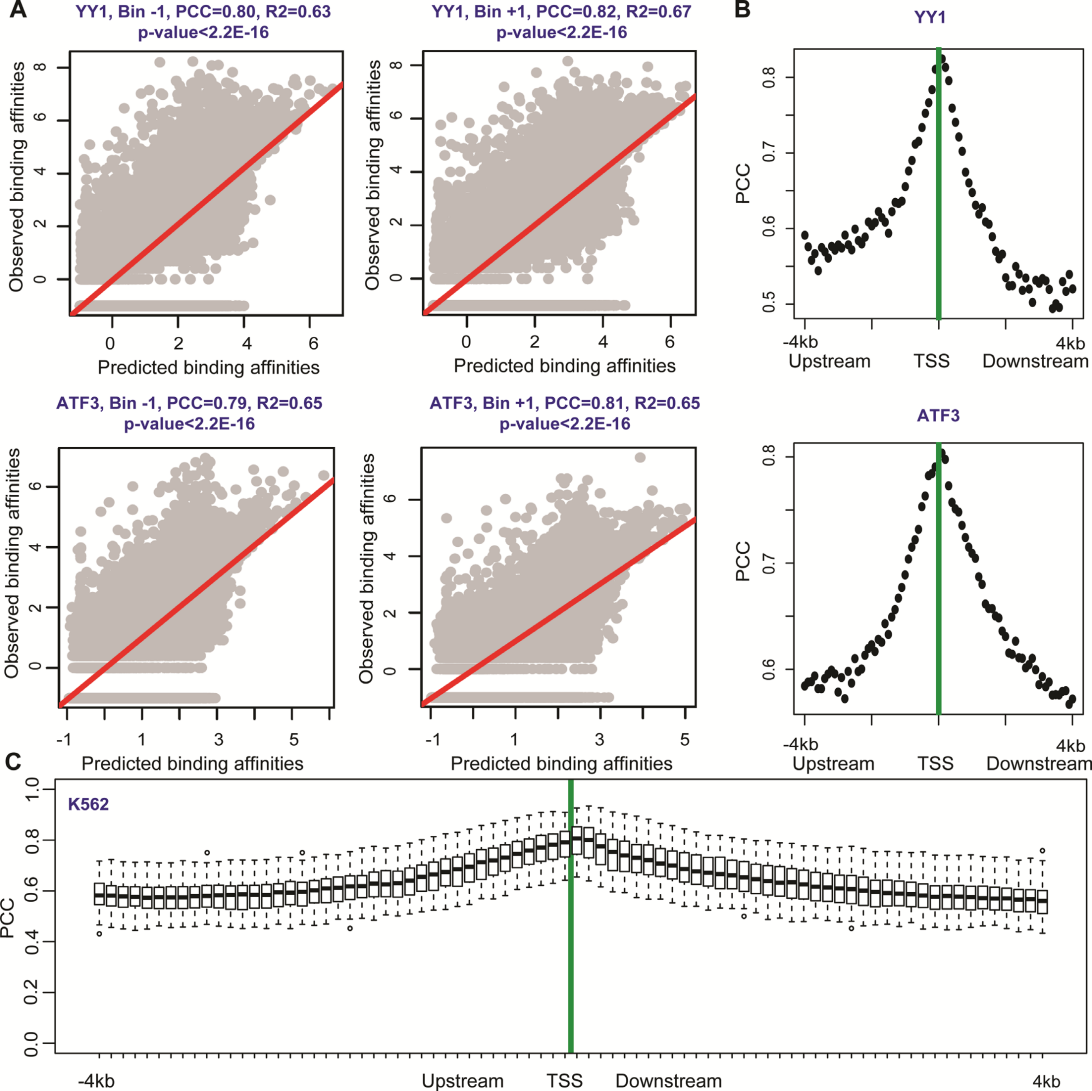
Comparisons between predicted TF binding affinities and experimental measurements from ChIP-Seq data. (**A**) The RF model was employed to predict the binding affinities of YY1 and ATF3 using histone modification and DNA methylation levels in upstream −100 to −1 bp (Bin −1) and downstream 1 to 100 bp regions (Bin +1). The linear regression line obtained from the ‘lm’ R package was shown in red. (**B**) Analyses for TF (YY1 and ATF3) predictions across 8k-bp regions centered at TSSs showed that epigenetic modifications were predictive to TF binding affinities, with the best predictions located in downstream regions (Bins +1 and 2). (**C**) Box plots for the prediction accuracies of binding affinities of 75 TFs with epigenetic modifications across 8k-bp regions in the K562 cell line. The RF model was employed in each bin for each TF. Generally high accuracies indicate that TF binding affinities can be precisely reflected by epigenetic modifications in any genome locations. The best (average) prediction accuracies are achieved in downstream 1 to 200 bp regions (Bins +1 and +2), and then predictions decreases in the two directions.

We aimed to study the quantitative relationships of epigenetic modifications to TF binding affinities. We employed computational approaches (e.g. the MLR and RF models) to study predicting ability of epigenetic modifications to TF binding affinities in each genomic region (bin) and the relative importance of each epigenetic feature (see ‘Materials and Methods’ section; Figure 1). As an example, within the upstream −100 to −1 bp region (Bin −1), the RF models achieved accurate predictions with PCC = 0.80 and PCC = 0.79 for YY1 and ATF3, respectively (Figure 2A). And within the downstream 1 to 100 bp region (Bin +1), similar accuracies were obtained for YY1 and ATF3 with PCC = 0.82 and PCC = 0.81, respectively (Figure 2A). We note that the use of pseudo-count −1 instead of 0 leads negligible changes in predictions (Supplemental Figure S1A). The MLR models were also built and tested, but gave poorer predictions, which indicates a non-linear relationship of epigenetic modifications with TF binding (Supplemental Figure S1B). The models were constructed and tested in all 80 bins. High prediction accuracies were observed (examples were shown in Supplemental Figure S2), with the best predictions for the two TFs occurring at the same downstream 0–200 bp regions (Bins +1 and +2), and prediction accuracies gradually decreasing in two directions (Figure 2B).

We performed this analysis using the RF models for all available TFs in each of 80 bins. Figure 2C and Supplemental Figure S3 show the prediction accuracies in the 80 bins centered at TSSs in the four human cell lines. Each figure contains various numbers of TFs, according to the availability of ChIP-Seq data (Supplemental Table S1). High prediction accuracies were achieved with average accuracy (PCC) reaching ∼0.64 across bins and TFs in each cell line, indicating epigenetic modifications can reflect TF binding affinities. The best predictions likely occur in the downstream 1 to 300 bp region (Bins +1 to +3), with median PCC reaching ∼0.80 and highest PCC = ∼0.93 in the four cell lines. Then prediction accuracies decay in the two directions as a function of increased distances to TSSs. Taken together, the high prediction accuracies across cell lines suggest the strong correlations between epigenetic modifications and TF binding affinities in all considered cell conditions.

### Contributions of epigenetic modifications change according to genome locations

Although epigenetic modifications regulate gene expression cooperatively, their contributions are not identical (17,49). Due to this fact, the correlations between each type of epigenetic modifications and TF binding affinities may change. We therefore studied the relative contribution of each epigenetic feature in each genome bin in order to detect the differences in the relationships between the two factors according to genome locations.

**Figure 3.**
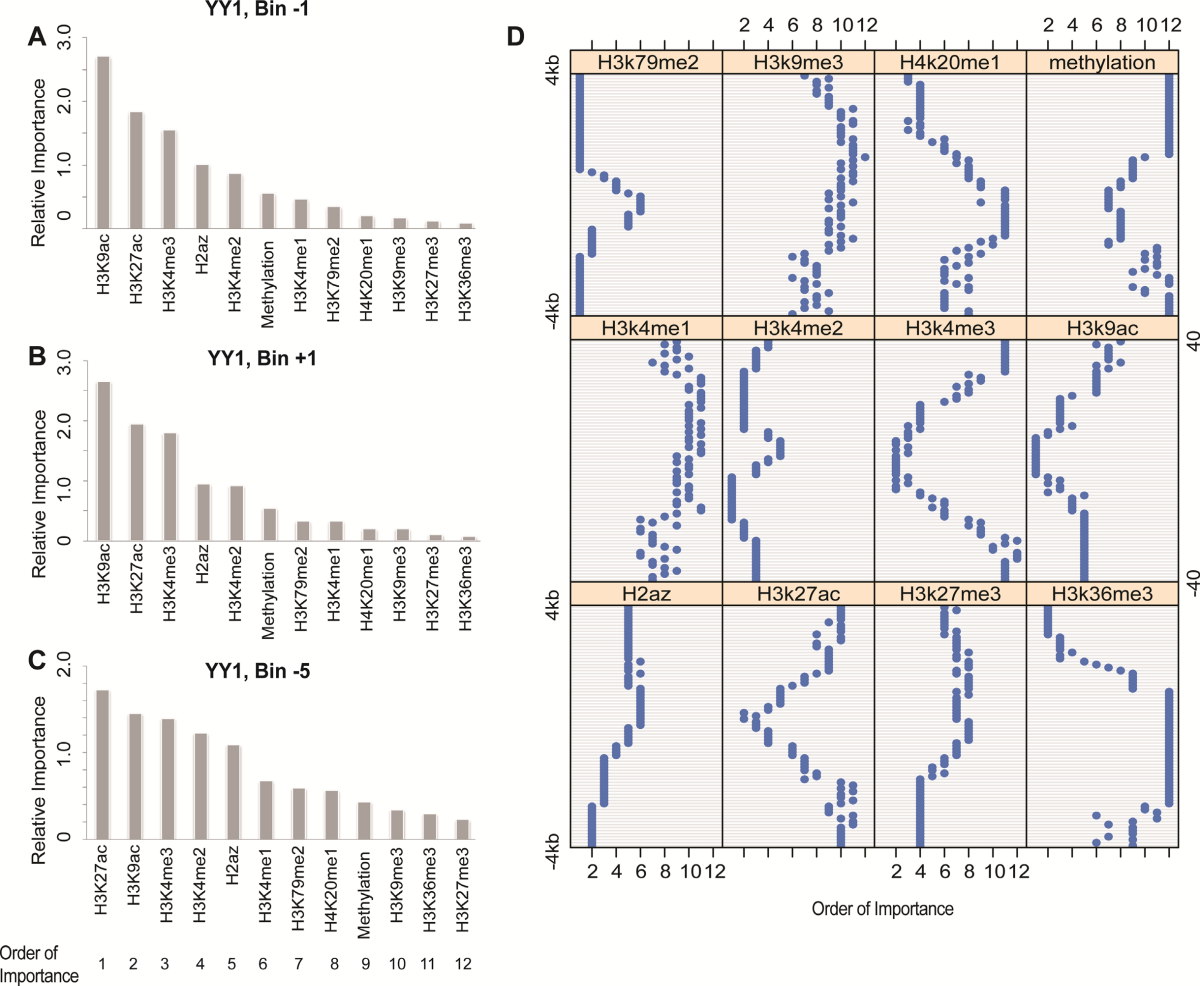
Relative importance of epigenetic modifications in predicting binding affinities of YY1 determined by their contributions to the prediction model. Different genome locations were plotted as examples: (**A**) −100 to −1 bp (Bin −1), (**B**) 1 to 100 bp (Bin +1) and (**C**) −500 to −400 bp regions (Bin −5). At each location, contributions from various epigenetic features are different, and the contribution patters change according to relative genome locations. The 12 types of epigenetic features were sorted and assigned as ‘order of importance’ from 1 to 12 in each bin based on their contributions to the prediction model. (**D**) The relative importance of epigenetic features in predicting binding affinities of YY1 across 80 bins centered at TSSs. Generally, H3K4me2, H3K27ac, H3k4me3 and H3k9ac play more important roles, in contrast to H3K9m3, H4k20me1 and H3k36me3, especially in the regions close to TSSs. DNA methylation is not top ranked, but plays relatively important roles in the regions closer to TSSs.

The relative importance of each epigenetic feature was obtained by analyzing its contribution to the aforementioned RF model in predicting TF binding affinities (see ‘Materials and Methods’ section). In the upstream −100 to −1 bp region (Bin −1), the six most important epigenetic modifications to binding affinities are H3K9ac, H3K27ac, H3K4me3, H2az, H3K4me2 and DNA methylation (Figure 3A for YY1; Supplemental Figure S4A for ATF3). The most important epigenetic modifications for YY1 in the downstream 1 to 100 bp region (Bin +1) are similar to those in the upstream region, but not for ATF3. H3K4me2 ranks above H2az, although their contributions are similar (Figure 3B; Supplemental Figure S4B).

When we analyzed the relative importance of epigenetic modifications across all 80 bins, a location-specific contribution of each epigenetic feature was observed. For example, in the upstream −500 to −401 bp region (Bin −5), the six most important epigenetic modifications for YY1 are H3k27ac, H3k9ac, H3k4me3, H3k4me2, H2az and H3k4me1; while for ATF3, the top six epigenetic modifications are H3K4me3, H3k9ac, H3k27ac, H3k4me2, H2az and H3k79me2, with DNA methylation ranked at ninth (Figure 3C; Supplemental Figure S4C).

Across all locations, H3K36me3 is generally not important in those regions close to TSSs (Figure 3D; Supplemental Figure S5), although its signal displays stronger (negative) correlations with TF binding affinities (Supplemental Figure S6B). Instead, H3K27ac, H3K9ac and H3K4me3, positively correlated with TF binding affinities (Supplemental Figure S6), are relatively important to TF binding at locations close to TSSs. DNA methylation plays important roles at locations near the TSSs, and decreases its contribution with increased distances from TSSs (Figure 3D; Supplemental Figure S5), in spite of the higher levels in these regions (Supplemental Figure S7). DNA methylation is observed to be more important than a few histone modifications, such as H3K36me3 and H3K9me3, which also have weaker correlations with TF binding affinities (Supplemental Figure S6). On the other hand, H3K4me1 and H3K4me2 are important in the locations far from TSSs (only exception for POLII in the HepG2 cell line). This result is supported by the reported correlations between gene expression and histone modifications, and the relative contributions of histone modifications in predicting gene expression (12,50).

Of note, the relative contribution patterns of epigenetic modifications generally exist across TFs and cell lines. After a comprehensive analysis in terms of genome locations, TFs, and cell lines, we found that H3K4me1, H3K4me2, H3k4me3 and H3k9ac are more likely to play important roles in TF binding predictions, in contrast to H3K27me3, H4k20me1 and H3k36me3, when considering average importance across bins and TFs (Supplemental Figure S8). This is consistent with the reported importance of chromatin features, such as H3K4me1, H3K4me2 and H3K4me3, for gene activity (4,13,14). The average contributions of epigenetic features change according to genome locations. H3K4me3, H3k9ac and H3k27ac are likely to be crucial when closer to TSSs, whereas H3K4me1 and H3k79me2 contribute more in the locations far from TSSs. Although DNA methylation is not top-ranked important, supported by previous researches that DNA methylation cannot solely select TFs for gene transcription (51,52), it displays a clear location-specific contribution pattern. When closer to TSSs, it contributes more than histone modifications such as H3k36me3, H4k20me1 and H3k9me3, while it is the least important feature in other locations. It is consistent with previously reported results on the association between DNA methylation and CTCF binding (53). All results illustrate the location-specific relationships between epigenetic modifications and TF binding.

### Epigenetic modification models for TF binding predictions are cell line-specific

**Figure 4.**
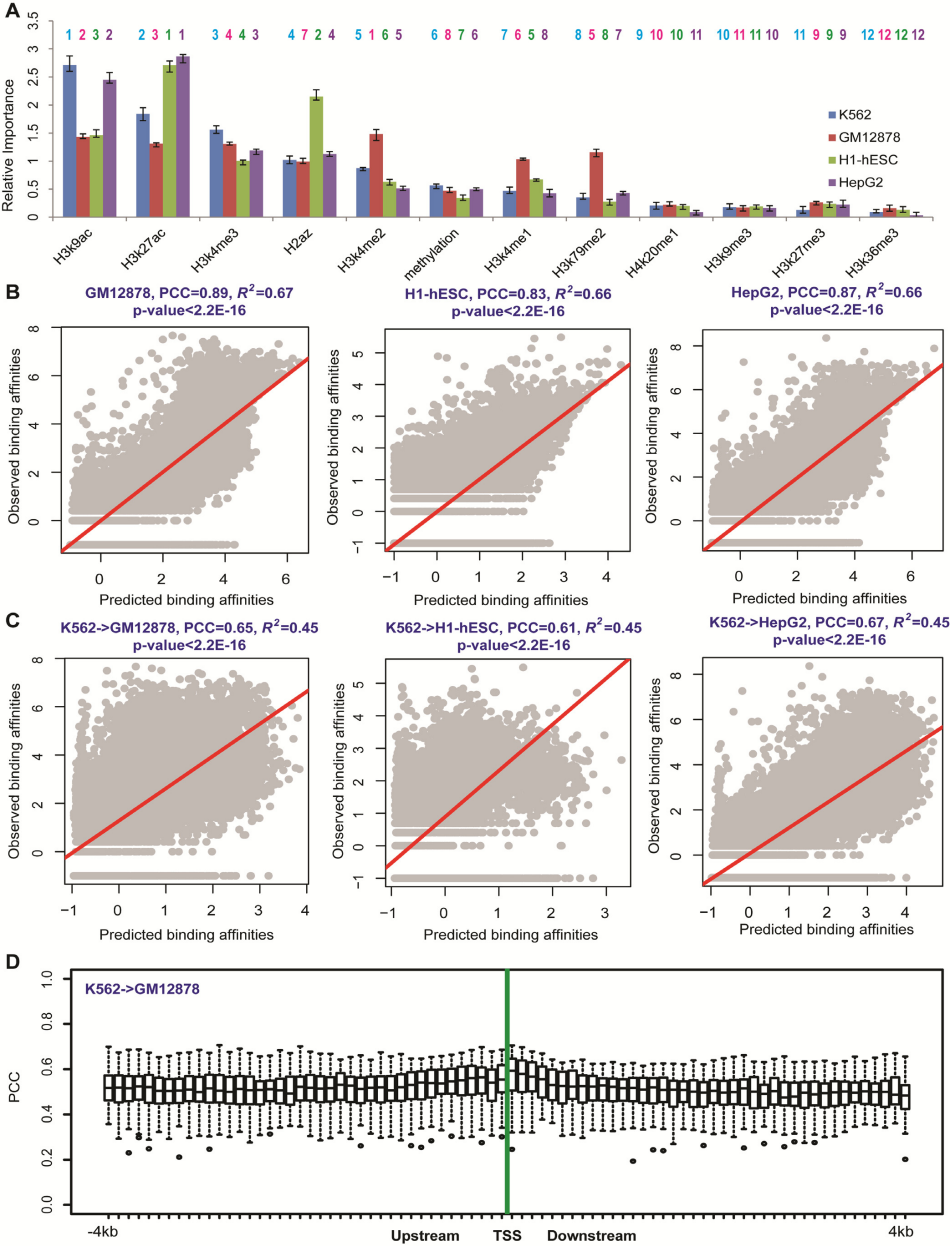
Epigenetic modification model for predicting TF binding affinity in each gene location is cell line-specific. (**A**) Relative importance of epigenetic modifications in predicting binding affinities of YY1 in the four (blue: K562, red: GM12878, green: H1-hESC and purple: HepG2) cell lines. The orders of relative importance of epigenetic features to model TF binding affinity across cell lines were shown on the top of each bar with corresponding colors. (**B**) Comparisons between predicted and observed binding affinities for YY1 within downstream 1 to 100 bp region (Bin +1) in the GM12878, H1-hESC and HepG2 cell lines. The RF models were trained and applied in the same cell line. (**C**) The epigenetic modification models learned from the K562 cell line were applied to other cell lines (GM12878, H1-hESC and HepG2) for predictions of YY1 binding affinities in downstream 1 to 100bp region (Bin +1). The epigenetic modification levels at the same genome location were used as inputs. (**D**) Box plots for the prediction accuracies of TF binding affinities with epigenetic modification models obtained from the K562 cell line and applied to the GM12878 cell line. 34 TFs were included due to the availability of ChIP-Seq data. In all 80 bins, prediction accuracies are clearly decreased, compared to the predictions with the models obtained in the same cell line, indicating that epigenetic modification models for TF binding predictions are cell line-specific.

Above analyses show that TF binding affinities can be predicted by epigenetic modifications in all considered cell conditions. However, contributions of epigenetic modifications in regression models for different cell conditions are distinct from each other (e.g. Figure 4A and Supplemental Figure S9A). This indicates that TF binding is related to epigenetic modifications in a cell type-specific manner (29,31–35,54,55). We further studied this cell line specificity of relationships between epigenetic modifications and TF binding affinities.

As shown in Figure 4A and Supplemental Figure S9A, the contribution of each epigenetic modification feature in predicting YY1 binding affinities (in Bins +1 and −1) changes with cell lines. We further applied the epigenetic modification models obtained from one cell line (K562) to others (GM12878, H1-hESC and HepG2), using the epigenetic modification levels in the same location (bin) as inputs, in order to explore whether the models can be generalized across cell lines (see ‘Materials and Methods’ section). As an example, the analyses within the downstream 1 to 100 bp (Bin +1) and the upstream −100 to −1 bp regions (Bin −1) show that prediction accuracies of 0.65, 0.61 and 0.67 (Figure 4C), and 0.71, 0.60 and 0.65 (Supplemental Figure S9C) in the GM12878, H1-hESC and HepG2 cell lines. These predictions are relatively lower than PCC = 0.89, 0.83 and 0.87 (Figure 4B), and 0.87, 0.82 and 0.87 (Supplemental Figure S9B), respectively. These results confirm the cell line specificity of epigenetic modification models.

Similar observations were obtained for other TFs and other bins. The epigenetic modification models were obtained from the K562 cell line in all 80 bins, and then applied to the other three cell lines (GM12878, H1-hESC and HepG2) using epigenetic modification levels in corresponding bin as inputs. Due to the fact that not all TFs were profiled by the ENCODE project, the numbers of available TFs in the four cell line are different. We included the TFs profiled in both the K562 and one of other three cell lines (Supplemental Table S1), that is, 34, 27 and 24 TFs in the GM12878, H1-hESC and HepG2 cell lines, respectively.

After applying the models constructed from the K562 cell line to the same TF in other three cell lines, the results show that prediction accuracies dramatically decreased, compared to predictions with models obtained in the same cell line (Figure 4C; Supplemental Figure S10). Average prediction accuracy (PCC) is reduced to ∼0.49, compared to 0.64 in each cell line. The best predictions still appear in the downstream 1 to 300 bp regions (Bins +1 to +3), while the median and highest prediction accuracies are decreased to ∼0.59 and 0.73, clearly lower than 0.80 and 0.93 when the models obtained in the same cell line were used. These results elucidate that epigenetic modification models for TF binding predictions are cell line-specific.

### Epigenetic modifications predict TF binding affinities genome-widely

Our analyses have shown the ability of epigenetic modifications to predict TF binding in the regions surrounding TSSs of protein-coding genes, where many TFs tend to cluster at (40,56–58). To comprehensively understand correlations between the two factors, we further tested our predictive approach in other regions, such as gene enhancers, which are usually far from TSSs (59,60) and suggested to consist of densely clustered TFBSs (56,58,61), and regions surrounding TSSs of non-protein-coding genes.

**Figure 5.**
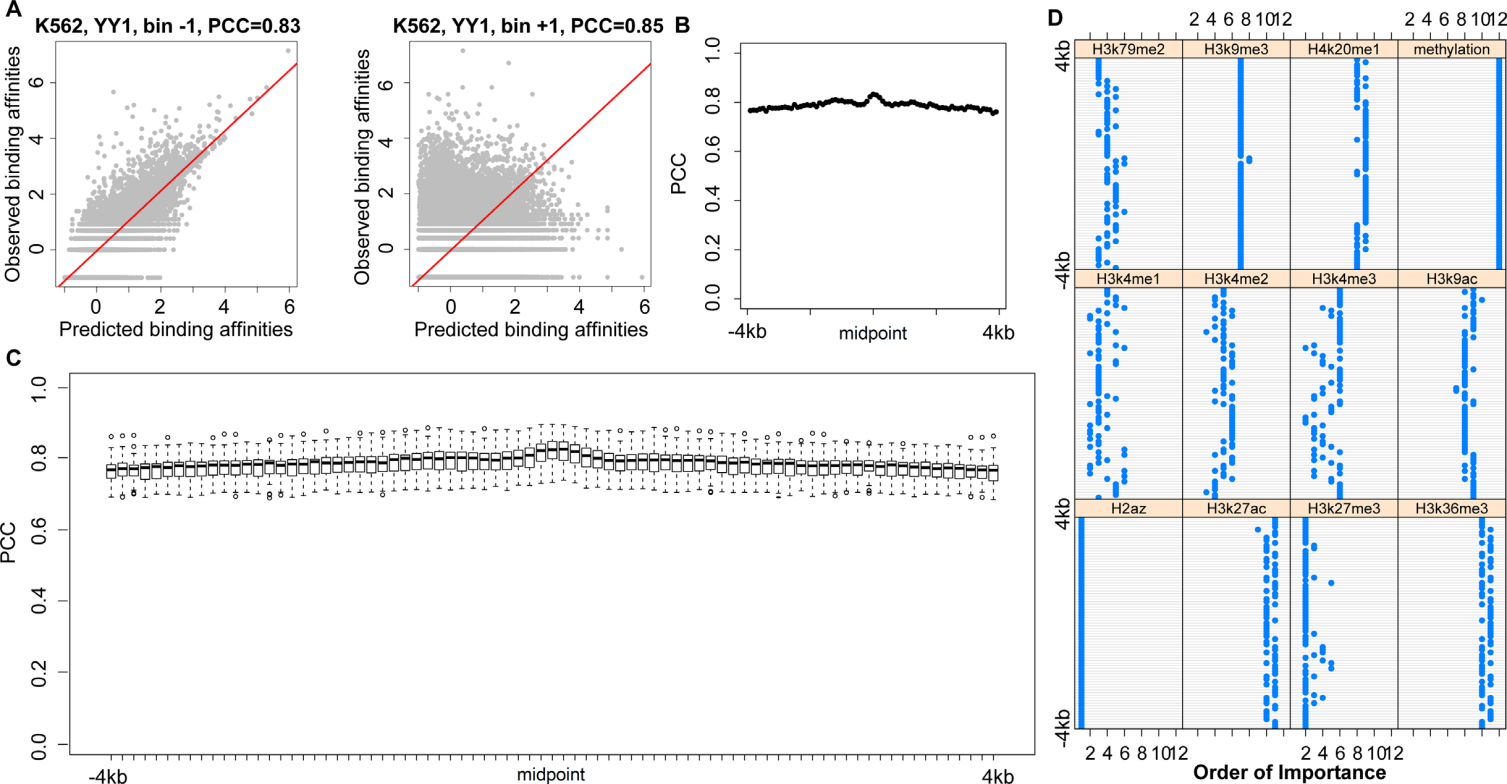
Performance of epigenetic modification models in upstream 4k-bp and downstream 4k-bp regions of gene enhancer midpoints. Prediction accuracies for YY1 in K562 cell lines were presented (**A**) in the regions of −100 to −1 bp (Bin −1) and 1 to 100 bp (Bin +1), and (**B**) across the 80 bins. (**C**) Prediction accuracies of all TFs in K562 cell lines. (**D**) Relative importance of epigenetic modifications in predicting YY1 binding affinities in K562 cell line.

Enhancers were defined by the FANTOM5 consortium (48). We considered the 8k-bp genome regions centered at the midpoint of each enhancer (Figure 1A). The RF models were rebuilt and applied to each bin with epigenetic features as inputs and TF binding affinities as outputs (Figure 1B). The predictions show that epigenetic modification signals are predictive to TF binding affinities in gene enhancers with high accuracies, e.g. PCC ∼ 0.80, for YY1 in K562 cell lines (Figure 5A and B) and for all TFs across the 8k-bp genome regions (Figure 5B; Supplemental Figure S11 for the GM12878, H1-hESC and HepG2 cell lines). Analyses on the contributions of epigenetic features to the RF models based on ‘%IncMSE’ values show that DNA methylation contributes the least in gene enhancer (Figure 5C). Instead, histone variant H2az plays the most important role, followed by H3K27me3, H3K4me1, H3K4me2 and H3K4me3.

**Figure 6.**
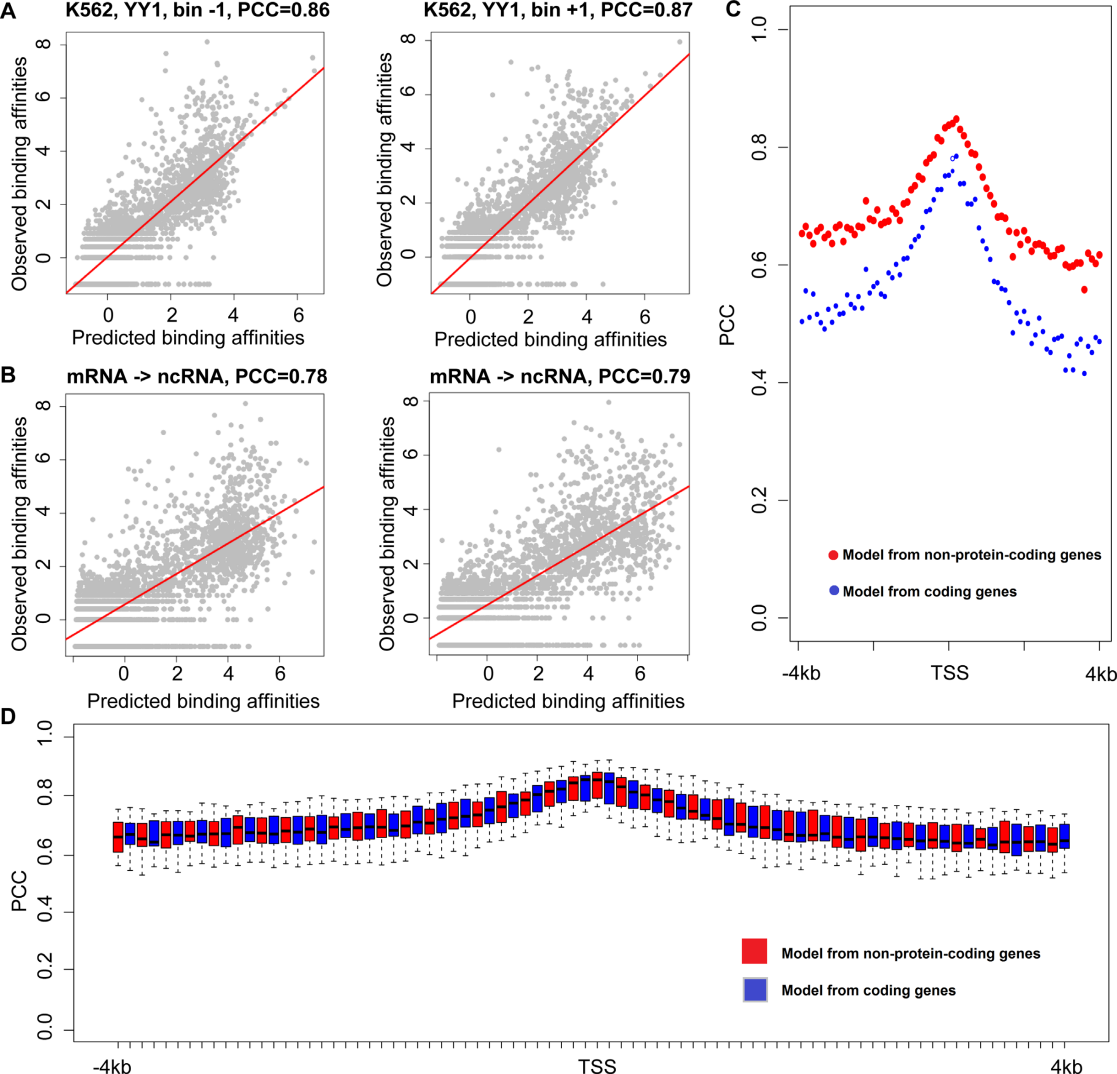
Epigenetic modification models for predicting TF binding affinities in non-protein-coding gene promoter regions. (**A**) Comparisons between predicted and observed binding affinities for YY1 within upstream −100 to −1 bp (Bin −1) and downstream 1 to 100 bp regions (Bin +1) in the K562 cell line. (**B**, **C**) The epigenetic modification models learned from protein-coding genes were applied to non-coding genes for prediction of YY1 binding affinities (B) in upstream −100 to −1 bp (Bin −1) and downstream 1 to 100 bp regions (Bin +1) in K562 cell line, and (C) across the 80 bins centered at TSSs. The epigenetic modification levels at the same genome location were used as inputs. (**D**) Box plots for the prediction accuracies of TF binding affinities with epigenetic modification models obtained from protein-coding genes and applied to non-coding genes. Red: epigenetic modification models were trained and applied to non-coding genes; Blue: epigenetic modification models were trained for protein-coding genes and applied to non-coding genes.

We also test the correlations in the regions surrounding TSSs of non-protein-coding genes. We first trained the RF models in regions (−4k to 4k-bp) centered at non-coding genes’ TSSs (Figure 1). For YY1 binding affinity predictions in K562 cell lines, the applications of this model achieved accuracies as 0.86 and 0.87 in bin −1 and bin +1, respectively (Figure 6A). We then tested the predicting ability of RF model trained for protein-coding genes to non-protein-coding genes. The results show that the prediction accuracies are reduced to 0.78 and 0.79 in the two bins, respectively (Figure 6B). Across the considered genome regions, prediction accuracies are generally reduced about PCC = 0.1 (Figure 6C; Supplemental Figure S12 for the GM12878, H1-hESC and HepG2 cell lines). For all TFs, similar results were discovered when models obtained for protein-coding genes were applied to non-protein-coding genes (Figure 6D). Taken together, these observations indicate that, epigenetic modifications are predictive of TF binding affinities genome-widely, although different patterns of histone modifications and TFs tend to appear at the promoters of protein-coding and non-coding genes or other regions (33,40,44,60,62–64).

## DISCUSSION

Regulation of gene expression by altering TF binding quantities and chemical modifications of DNA sequence is a fundamental mechanism. Certain relationships between TF binding and epigenetic modifications have been discovered. When studying these correlations, researches have only focused on the influence of epigenetic modifications on TF binding sites or motifs (5,31,41,49,65,66). Our model enables a systematic analysis of correlations between the two factors in a quantitative manner. Also we revealed that the correlations vary according to genome locations and cell types.

We analyzed the correlations in both TF binding peak and non-peak genome regions. The latter were normally considered less important and ignored in previous peak-based studies. As a fact, although non-peak regions contribute less in the study of overall TF binding motifs or binding sites, it is required to investigate the correlations in these regions for a comprehensive understanding. Lower binding quantities may be related to different contributions of DNA sequence or various epigenetic modifications. By dividing genome regions into bins, our model enables to study the genome-wide correlations between epigenetic modifications and TF binding, and to discover the location-associated relationships quantitatively.

Our method benefits the study of histone modification and DNA methylation status, and their direct correlations with TF binding. The profiles of histone modifications usually show very broad peaks across genome and are not convenient to be analyzed with the peak-based method. By representing levels with average coverage of 100 nucleotides in each bin, we can locally evaluate histone modification levels and their direct correlations with TF binding. For DNA methylation, available methods considered the CpG sites within a certain distance from TF binding peaks (e.g. (28,53,67)). Instead, our method enables to study direct correlations of DNA methylation to TF binding.

We confirmed that, even without depending on sequence information, epigenetic modifications are predictive of TF binding affinities, and besides specific regions, such as enhancers (68), correlations between epigenetic modifications and TF binding exist in all genome regions. These correlations do not happen by chance, but are raised from the naturally biological mechanisms (see Supplemental Materials, and Supplemental Figure S13). TFs can account for around 10% of genes in human, representing the largest family of proteins (69). These TFs may interact with DNA sequences across the whole genome with specific sequence motifs and binding patterns. Chemical/epigenetic modifications to genomic sequences and nearby histones may also happen at most genome locations (3,19,20). These changes can affect gene transcription from initiation to elongation and termination, and consequently overall gene expression. For example, H3K4me1/2/3 mainly exist in promoter regions and promote transcription initiation, while H3K36me3 contributes to transcription elongation in transcribed regions.

Our analyses illustrated strong correlations between epigenetic modifications and TF binding affinities, since the former is informative for accurate predictions of the latter. We further validated the generality of these correlations. For example, previous studies indicated that TF binding affinities are highly dependent on expression levels of genes that are bound by the TF (70,71). We tested if our predictions are changed according to gene expression levels. Results show that our predicting model is generally valid for all genes with similar prediction accuracies, regardless of their expression levels (see Supplemental Materials, and Supplemental Figure S14). Moreover, to test whether the correlations generally exist across the whole genome, we extended the testing regions to upstream 10k bp of protein-coding genes. In this region, our results also illustrate the strong correlations between the two factors by achieving high prediction accuracies with epigenetic modification models (see Supplemental Materials, and Supplemental Figure S15A and B).

Taken together, the genome regions considered in this work cover both TF-clustered and non-TF-clustered genome sequences. TFs tend to regulate gene expression through a combination of physical mechanisms (56,72–74). As well, previous studies of ChIP-Seq analyses showed that 75% of TF peaks are localized in only 0.8% of the genome, and each TF-clustered region is less than 2k bp (56,75). These TF-binding sequences may locate in the regions near TSSs (promoters) (40,56–58) or at a distance of thousands of bps away from TSSs (enhancers) (56,58), with the former spanning less than 2k-bp genome sequence (76,77) and the latter ranging between 50 and 1500 bp (78,79). The lengths of enhancers can also be obtained with the information from the FANTOM5 consortium (48) (Supplemental Figure S16). Our analyses also illustrated that predictions may vary among functional regions of the genome, e.g., epigenetic modification models achieved significantly better predictions in gene enhancers than in promoters (Supplemental Figure S17).

Epigenetic features can reflect the genome-wide correlations between the two factors in any considered cell conditions, but the prediction models are cell line-specific. Previous peak-based studies have discovered that the usage of regulatory elements for TF binding displays a cell type-specific fashion. This specificity is associated with DNA sequence (31) and one or more chromatin alternations, including histone modifications (32–35), DNA methylation status (29,36), accessibility of regulatory elements (31,80,81) and chromatin conformation and DNA looping (54). Our model enables to analyze this type of correlations by considering both histone modifications and DNA methylation. Moreover, models for different cell lines are not interchangeable, indicating cell type-specific correlations between TF binding and epigenetic modifications.

Our analysis elucidated that epigenetic features work in a combinatory and non-linear fashion to reflect the TF binding affinities, and implied that only a few histone modifications are necessary to faithfully model TF binding at each genome location. This is supported by experimental evidences, e.g. certain histone modifications, such as H3K4me2 and H3K4me3, but not all the H3 ones, are crucial to TF binding (82,83), and can work as marks for transcriptional activity (4,13,14). This result also agrees with previous findings about contributions of histone modification to gene expression variants (49). Our model further illustrated that contributions of epigenetic modifications may change according to genome locations and cell lines/conditions, indicating the location- and cell line-specific associations (31,54).

We proved that, although DNA methylation does not play dominant roles in modeling of TF binding, it has a relatively higher contribution than a few types of histone modifications, especially in the regions near TSSs. The relative importance of DNA methylation generally decreases with an increase of distance, which is consistent with previously reported association between DNA methylation level and CTCF binding (53).

**Figure 7.**
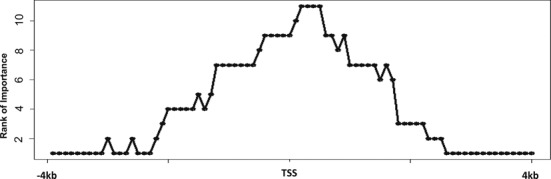
Relative importance of nucleosome occupancy across the 80 bins centered at TSSs for predicting YY1 binding affinities in K562 cell line.

The model can be extended by including other epigenetic or chromatin structure information. For example, genome regions that are more strongly bound by TFs are flanked by better-positioned nucleosomes (44), and nucleosomes are suggested to be gatekeepers of TFBSs (64). The consideration of nucleosome positioning data will be helpful for understanding the relationship between chromatin structure and TF binding. Our analyses show that prediction accuracies (PCCs) are ∼0.02 higher when including nucleosome positioning data, and nucleosome occupancy is not top ranked in the prediction model when close to TSSs (see Supplemental Materials, and Supplemental Figures S18 and S19), but more important when away from TSSs (Figure 7).

## SUPPLEMENTARY DATA

Supplementary Data are available at NAR Online.

SUPPLEMENTARY DATA
