# Supplementary material for: Modeling the relationship of epigenetic modifications to transcription factor binding
Source: Nucleic Acids Res. 2015 Mar 27;43(8):3873–85. doi: 10.1093/nar/gkv255 (PMC4417166; doi:10.1093/nar/gkv255)
Supplement: SUPPLEMENTARY DATA [file supp_43_8_3873__index.html]

Modeling the relationship of epigenetic modifications to transcription factor binding — SUPPLEMENTARY DATA 

# Modeling the relationship of epigenetic modifications to transcription factor binding

## SUPPLEMENTARY DATA

**Files in this Data Supplement:**

- SUPPLEMENTARY DATA
