# Supplementary material for: Modeling the relationship of epigenetic modifications to transcription factor binding
Source: Nucleic Acids Res. 2015 Mar 27;43(8):3873–85. doi: 10.1093/nar/gkv255 (PMC4417166; doi:10.1093/nar/gkv255)
Supplement: SUPPLEMENTARY DATA [file supp_gkv255_nar-03456-z-2014-File009.docx]

**Modeling the relationship of epigenetic modifications to transcription factor binding**

Liang Liu^1^, Guangxu Jin^1,*^, and Xiaobo Zhou^1,*^

^1^Center for Bioinformatics & Systems Biology and Department of Radiology, Wake Forest School of Medicine, Winston-Salem, NC 27157, USA

* To whom correspondence should be addressed. Tel: +1-336-713-1789; Fax: +1-336-713-5891; Email: [xizhou@wakehealth.edu](mailto:xizhou@wakehealth.edu)

* Correspondence may also be addressed to G.J. Tel: +1-336-716-6977; Email: [gjin@wakehealth.edu](mailto:gjin@wakehealth.edu)

**SUPPLEMENTAL MATERIALS**

1. **Supplemental Materials**
2. **Supplemental Figures S1-S19**
3. **Supplemental Table S1**

**1. Supplemental Materials**

**Validation of predicting model**

To assure the reliability of our predicting model, and validate the correlations between epigenetic modifications and TF binding affinities, we shuffled the pairing between TF binding and epigenetic modifications (e.g. using epigenetic modifications of gene 2 to predict TF binding on gene 1). The predictions were reduced to very low accuracies. For example, in the bins close to TSSs, prediction accuracies are reduced to ~0.01 (Supplemental Figure S13A), significantly lower than the accuracies, 0.80 and 0.82, shown in Figure 2A. We repeated this process for 50 times, and the maximum prediction accuracy is only ~0.025 (Supplemental Figure S13B). This test proves that correlations between the two factors do not happen by chance, but are raised from the naturally biological mechanisms.

**Epigenetic models are valid for all genes**

Previous studies have suggested that TF binding affinity is highly dependent on expression level of the gene that is bound by the TF ([1](#_ENREF_1),[2](#_ENREF_2)). Therefore, to test whether the predicting models are generally valid, and whether the observed correlations between epigenetic modifications and TF binding affinities are biased by highly expressed genes, we categorized the RefSeq genes into groups and tested the prediction ability.

We downloaded the expression profiles from the ENCODE project, which were performed with Affymetrix Human Exon 1.0 ST Array platform (GEO access number: [GSE1580](http://www.ncbi.nlm.nih.gov/geo/query/acc.cgi?acc=GSE15805)5), and then analyzed expression levels for protein-coding RefSeq genes. We then categorized them into four groups based on their expression quartiles (Q1, 2, 3 and 4).

Epigenetic modification models were constructed for each group of genes and in each genomic bin. We found that prediction accuracies for these four groups are similar. For example, Supplemental Figure S14A shows prediction accuracies in bin +1 (downstream 1 to 100bp, YY1 in K562 cell line) are all around 0.80, which is close to the prediction accuracy when all genes were treated as one database (PCC = 0.82, Figure 2A). Across the 80 bins, the prediction accuracies are very close as well (Supplemental Figure S14B). These observations illustrate that, although the binding affinities of TFs may be dependent on the expression level ([1](#_ENREF_1),[2](#_ENREF_2)), our models are generally valid for all genes with similar prediction accuracies.

**Epigenetic modification models in upstream 10k to 4k-bp regions**

We extended the genome regions to upstream 10k to 4k bps of protein-coding gene TSSs, and also divided these regions into 80 bins. The RF model was trained in each bin for each TF. Examples are shown in Supplemental Figure S15. Predicting abilities of the epigenetic modification models in this region do not vary significantly, with accuracies around PCC=0.6 (Supplemental Figure S15A and B). The contributions of epigenetic features do not vary as well (Supplemental Figure S15C), and keep consistence with previous findings (Figure 3D).

**Consideration of nucleosome positioning data**

Nucleosome positioning data have been generated with the MNase digestion and high-depth sequencing in the GM12878 and K562 cell lines by Kundaje et. al ([3](#_ENREF_3)). Similar to histone modifications, we calculated the coverage of 80 bins centered at TSSs and normalized by the library size. Then we combined this data with other epigenetic modifications to construct and test the model.

The inclusion of nucleosome positioning data increases the prediction accuracy very moderately. For example, for YY1 in K562 cell line, prediction accuracies were increased from 0.80 and 0.82 (Figure 2A) to 0.81 and 0.84 in the Bins -1 and +1, respectively (Supplemental Figure S18A). Across the 80 bins, minor changes of accuracies were also observed (Supplemental Figure S18B).

In addition, we checked the importance of nucleosome positioning in the RF model for binding affinity prediction. We found that nucleosome occupancy are not top ranked in the regions closing to TSSs (Supplemental Figure S19A and B), but play important roles when away from TSSs (Supplemental Figure S19B).

**2. Supplemental Figures S1-S19**

**
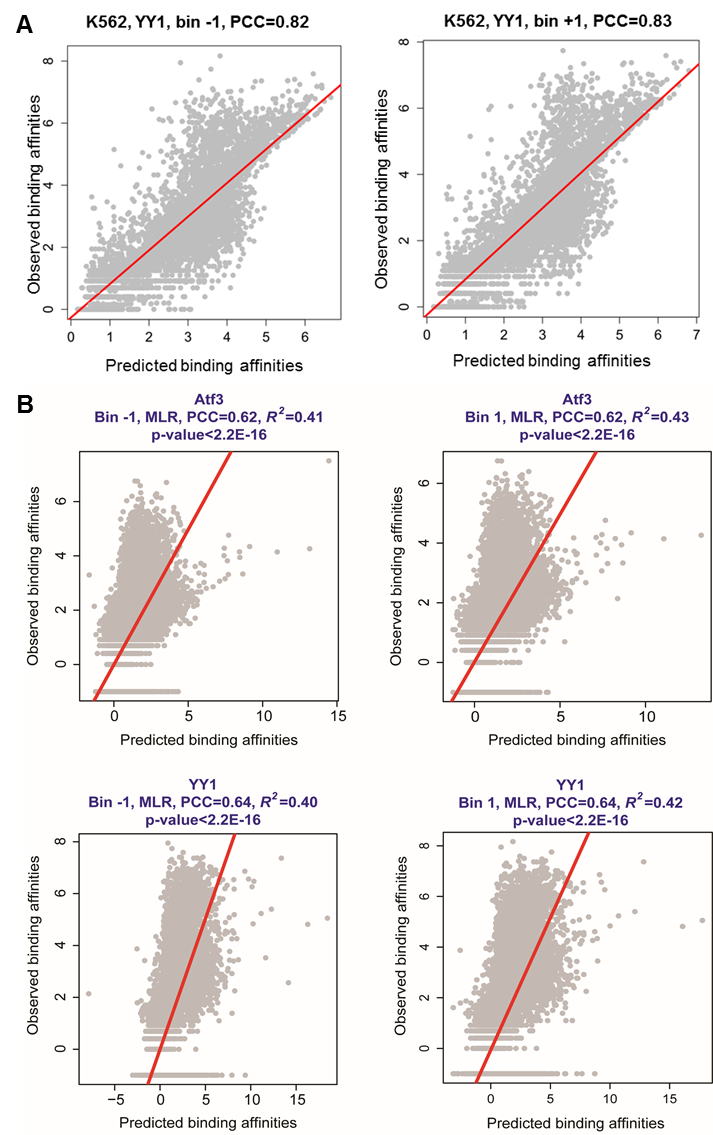
**

**Supplemental Figure S1:** Comparisons between predicted TF binding affinities and experimental measurements from the ChIP-Seq data. (A) The RF models were built and tested with binding affinities of the bin set to 0 instead of pseudo-count -1 when no ChIP-Seq reads were mapped to bin. (B) The MRL models were employed to predict the binding affinities of YY1 and ATF3 using histone modification and DNA methylation levels in upstream -100 to -1bp (Bin -1) and downstream 1 to 100bp regions (Bin +1).

**
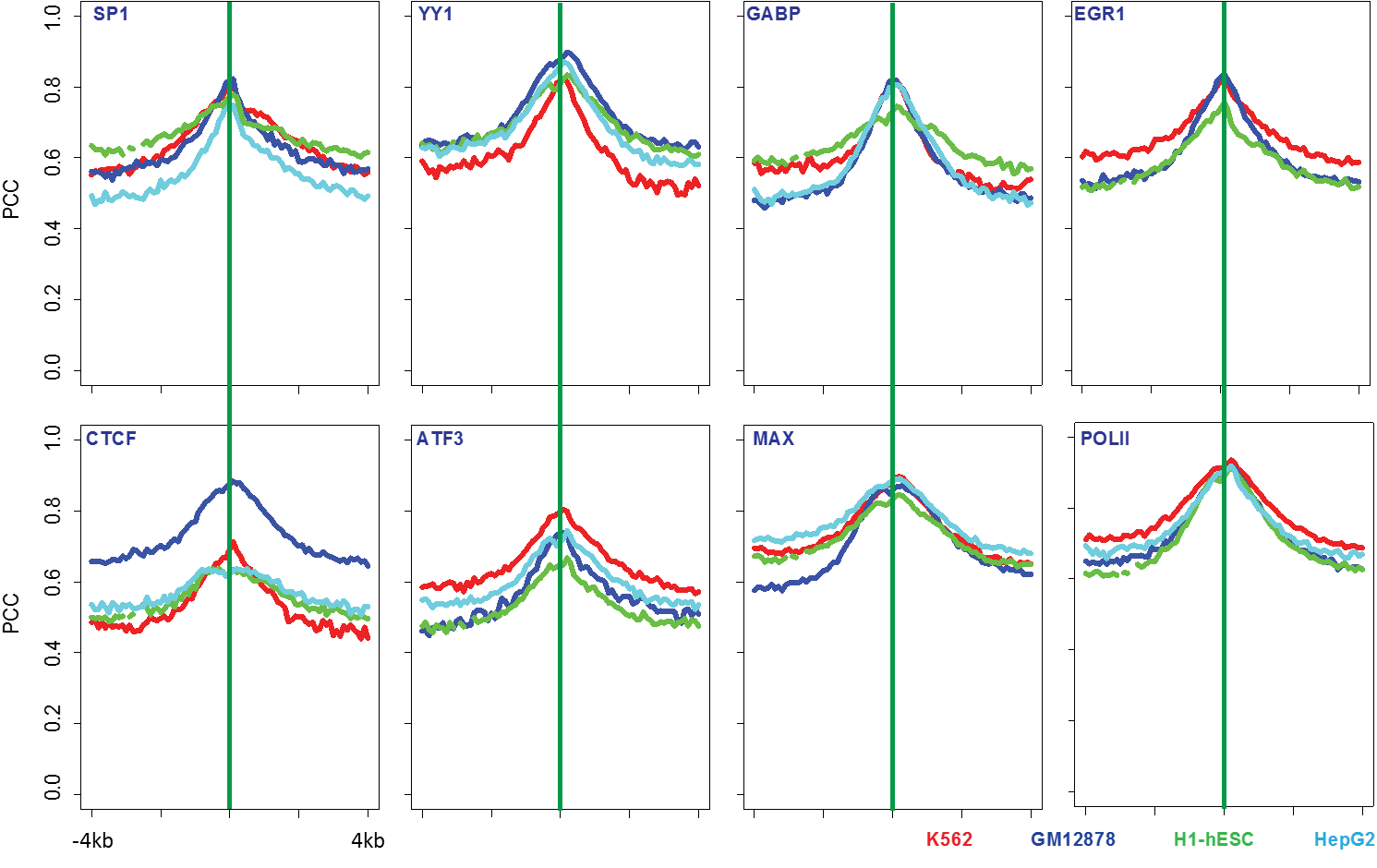
**

**Supplemental Figure S2:** Comparisons between TF binding affinities predicted by epigenetic modification models and observed values in regions surrounding TSSs in the four cell lines. Red: K562; Blue: GM12878; Green: H1-hESC; Cyan: HepG2. Plots are incomplete due to availability of data (e.g., HepG2 cell line does not have ChIP-Seq data for EGR1 and MAX). The predictions achieved high accuracies overall with the best PCC being ~0.90. Accuracy decays with an increase of distance to TSSs.

**
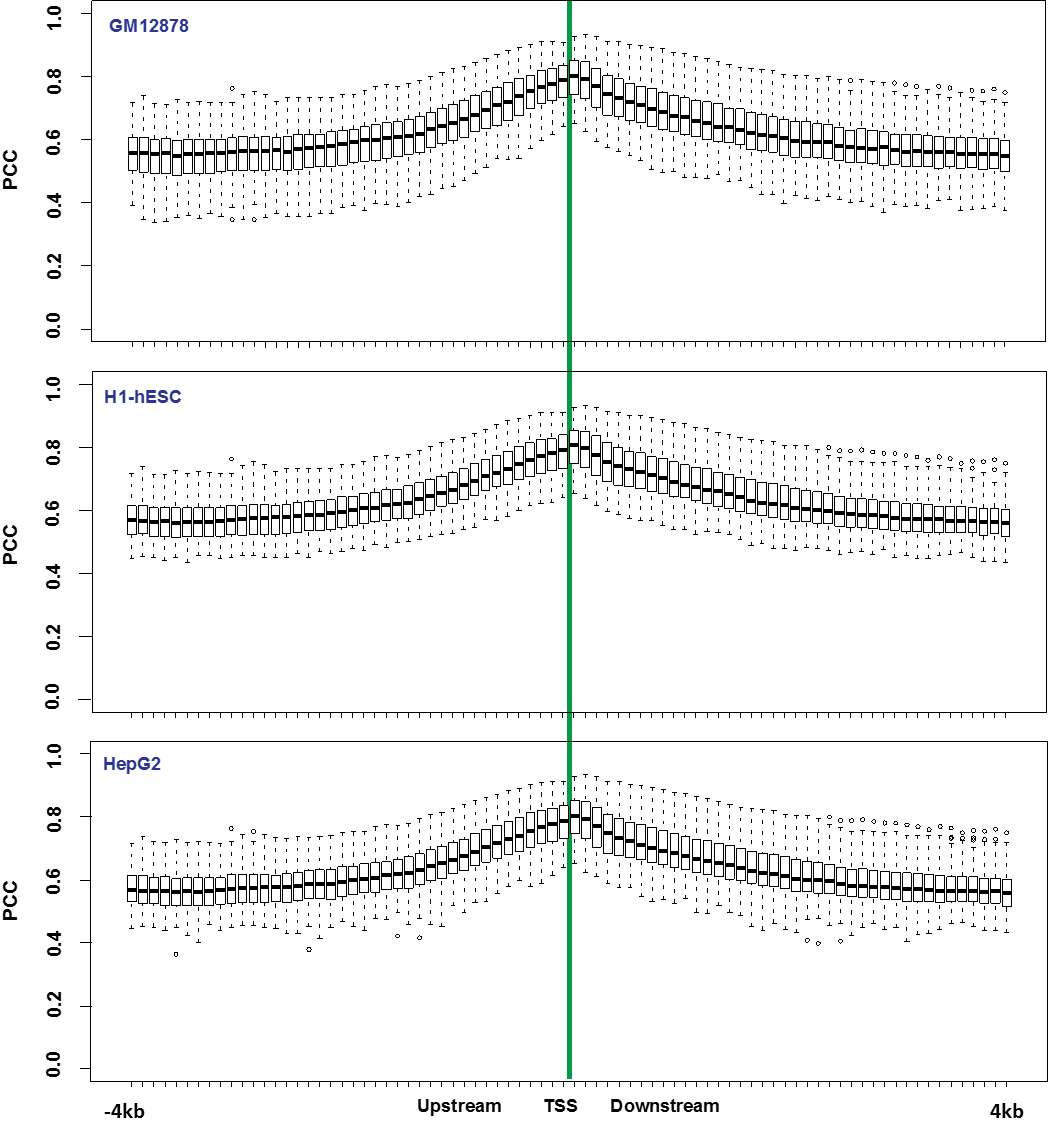
**

**Supplemental Figure S3:** Box plots for prediction accuracies of TF binding affinities with epigenetic modifications across 8k-bp regions in the GM12878, H1-hESC, and HepG2 cell lines. The RF model was employed in each bin for each TF and cell line. Various numbers of TFs were included in each figure due to the availability of ChIP-Seq data (Supplemental Table S1). High accuracies indicate that TF binding affinities can be precisely reflected by epigenetic modifications in any cell condition. The best (average) prediction accuracy over all TFs is achieved at downstream 1 to 300bp regions (Bins +1, +2 and +3), and then predictions decrease in the two directions.

**
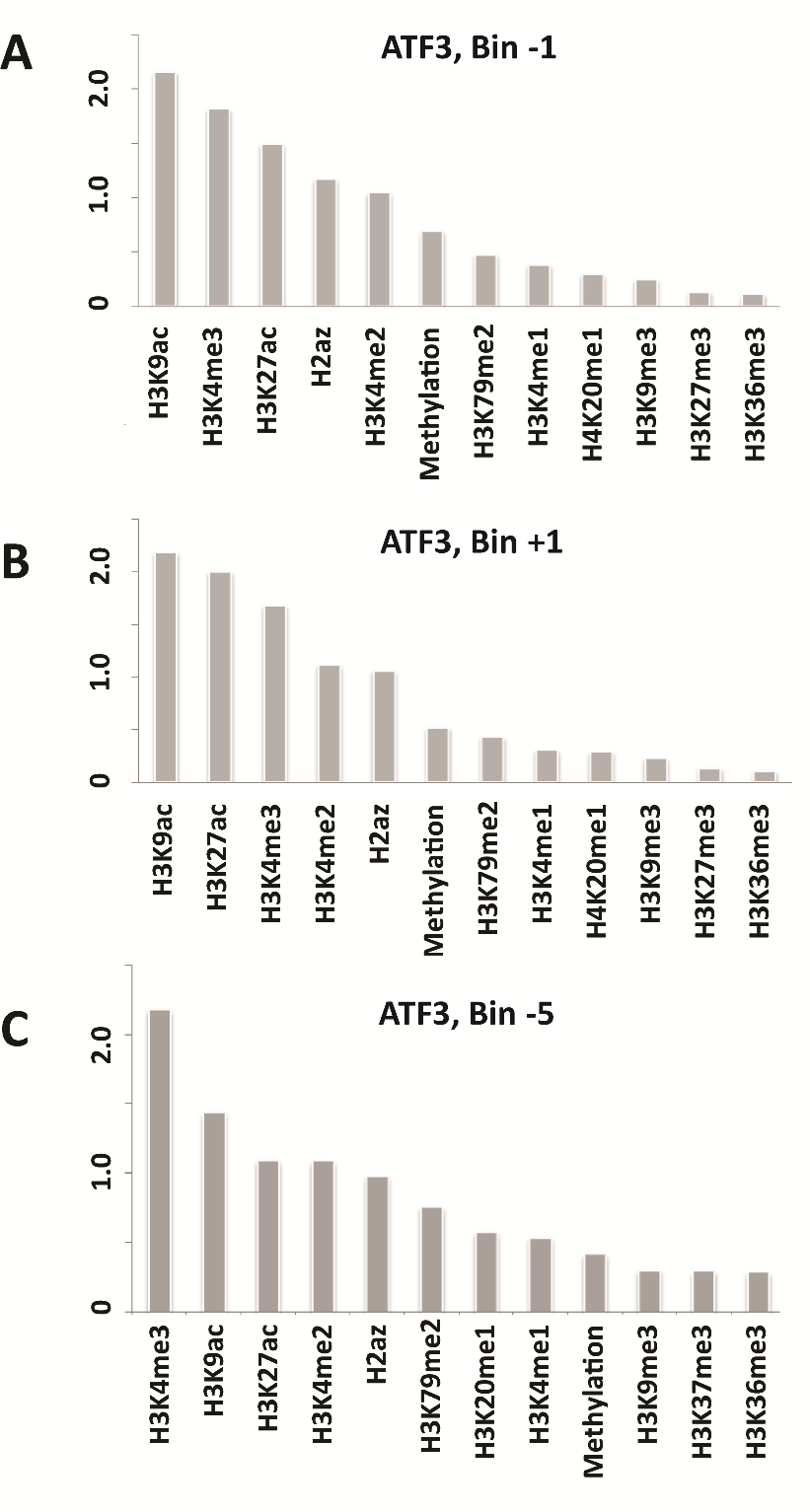
**

**Supplemental Figure S4:** Relative importance of epigenetic modifications in predicting binding affinities of ATF3. Differently relative genome locations were plotted as examples: (A) -100 to -1bp (Bin -1), (B) 1 to 100bp (Bin +1) and (C) -500 to -400bp regions (Bin -5). In each bin, contributions from various epigenetic features are different, and the contribution patters change according to relative genome locations.


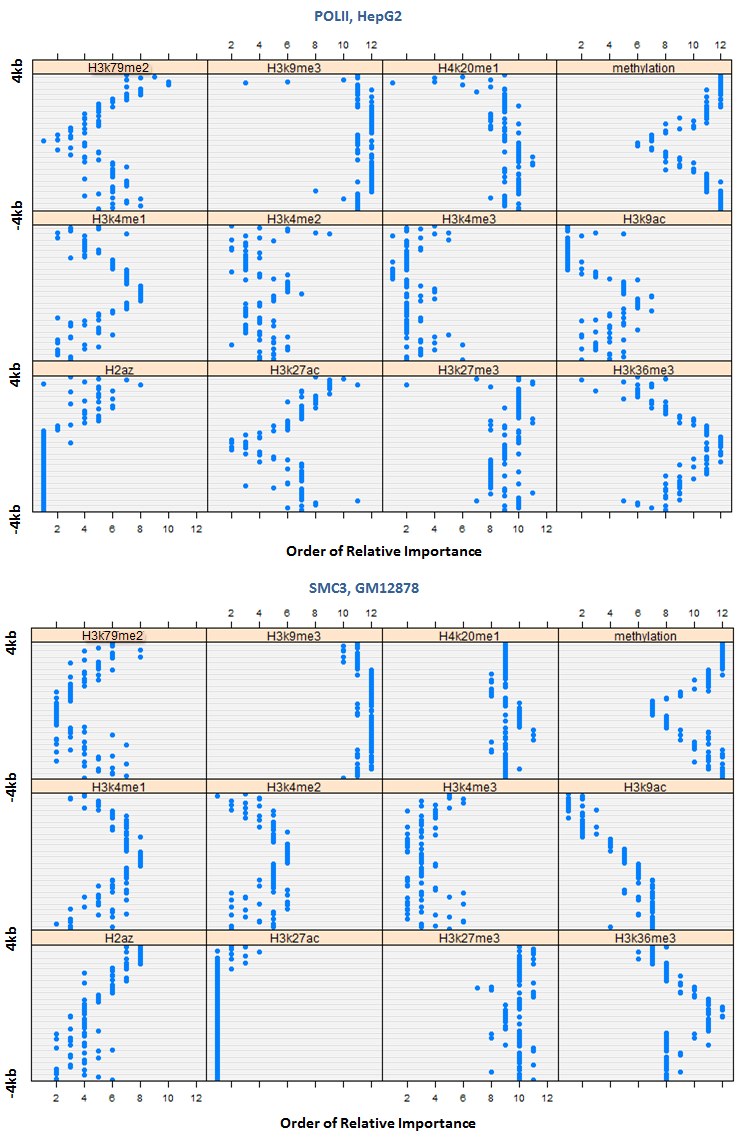


**
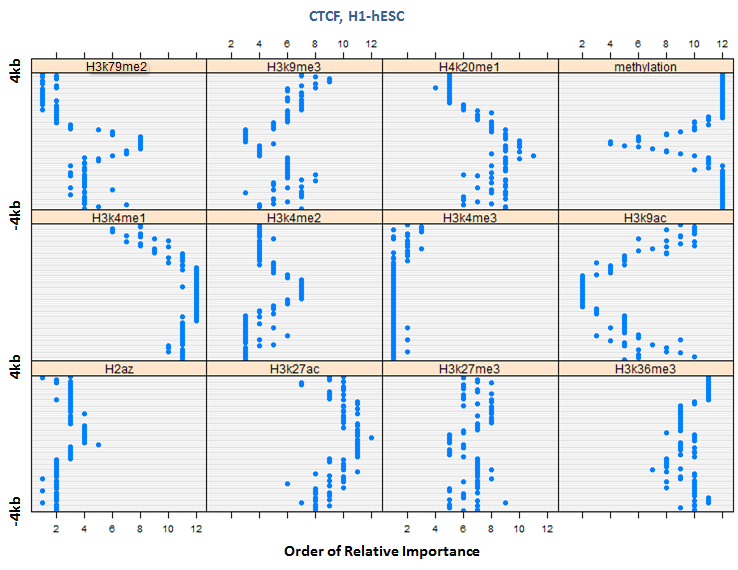
**

**Supplemental Figure S5:** Relative importance of epigenetic modifications in predicting TF bindings in the RF model. POLII, SMC3 and CTCF in the HepG2, GM12878, and H1-hESC cell lines, respectively, were chosen as examples. Only a few histone modifications are important to the modeling of TF binding, while DNA methylation contributes more than some of histone modifications, especially in regions neighboring TSSs. The contribution of each epigenetic modification displays a location-specific pattern.


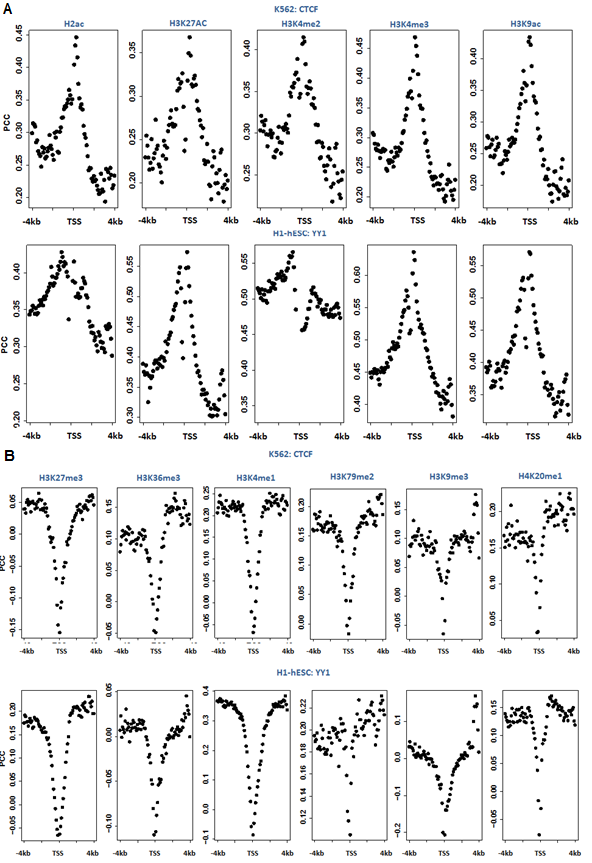


**Supplemental Figure S6:** Correlation (PCC) patterns between TF binding affinities and histone modification levels around TSSs across cell lines. The genes or bins without measured TF binding affinity (assigned with -1) were excluded. Correlations between histone modifications and TF binding affinities vary according to chromatin features. (A) Histone modifications (including H2az, H3K27ac, H3K9ac, H3K4me2 and H3K4me3) are positively correlated with TF binding, with correlations (PCC) decreasing as distance from TSSs increased. (B) Other histone modifications have moderately negative correlations with TF binding at TSSs. For H3K4me1, although a slightly negative correlation is observed at TSSs (e.g., PCC ~ -0.05 and -0.1 in K562 and H1-hESC cell lines, respectively), positive correlations are found in other regions.


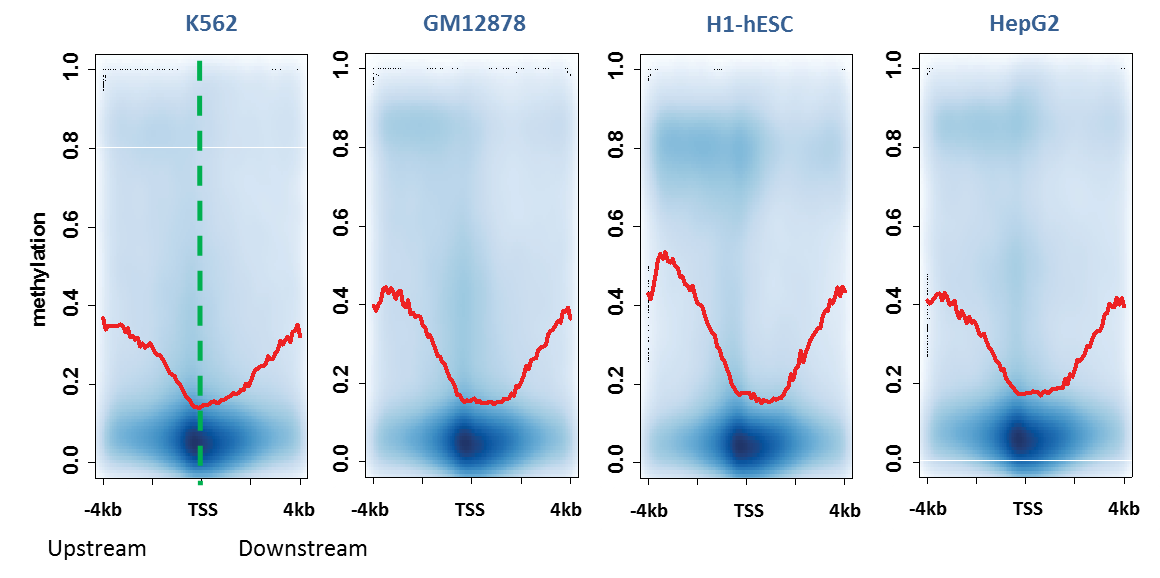


**Supplemental Figure S7:** Density plots of DNA methylation levels surrounding TSSs of the RefSeq genes. The average levels over all genes in each bin are represented with red lines across 8k-bp regions. DNA methylation level tends to be lower in regions closer to TSS than other regions.


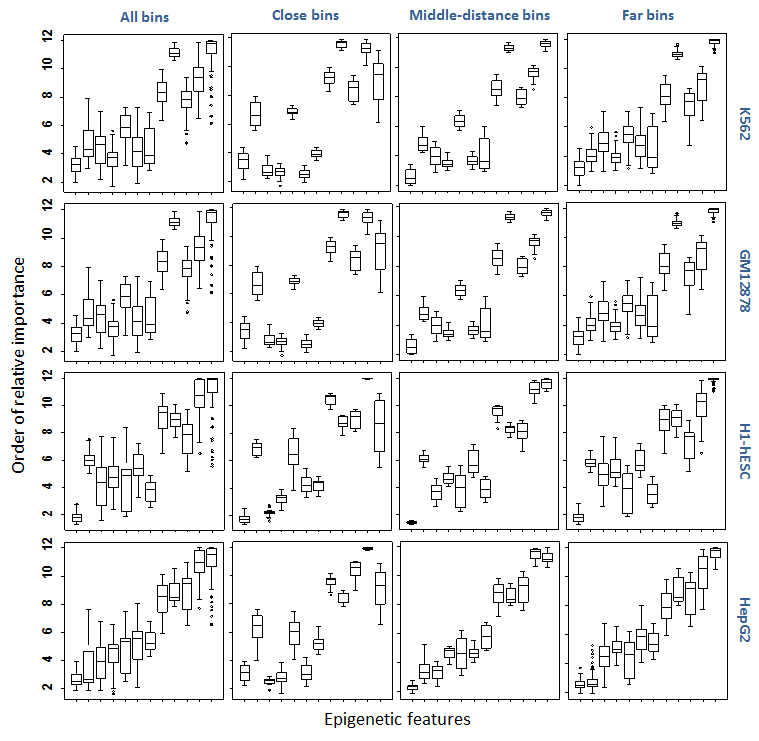


**Supplemental Figure S8:** The average relative contribution orders of epigenetic modifications to predicting TF binding affinities in the four human cell lines. Average was taken over (all, close, middle-distance and far) bins and TFs in each cell line. Different groups of bins were defined as: all region, all the 80 bins; close bins, -1000 to 1000bp regions (Bins ±1 to ±10); middle-distance bins, -2000 to -1001bp and 1001 to 2000bp regions (Bins ±11 to ±20); and far bins, -4000 to -2001bp and 2001 to 4000bp regions (Bins ±21 to ±40). Epigenetic features in each figure are listed as (from left to right): H3k4me2, H3k4me1, H3k4me3, H3k9ac, Hek79me2, H3k27ac, H2az, H3k9me3, H3k27me3, H4k20me1, H3k36me3 and DNA methylation. Contributions of epigenetic features change according to genome locations.


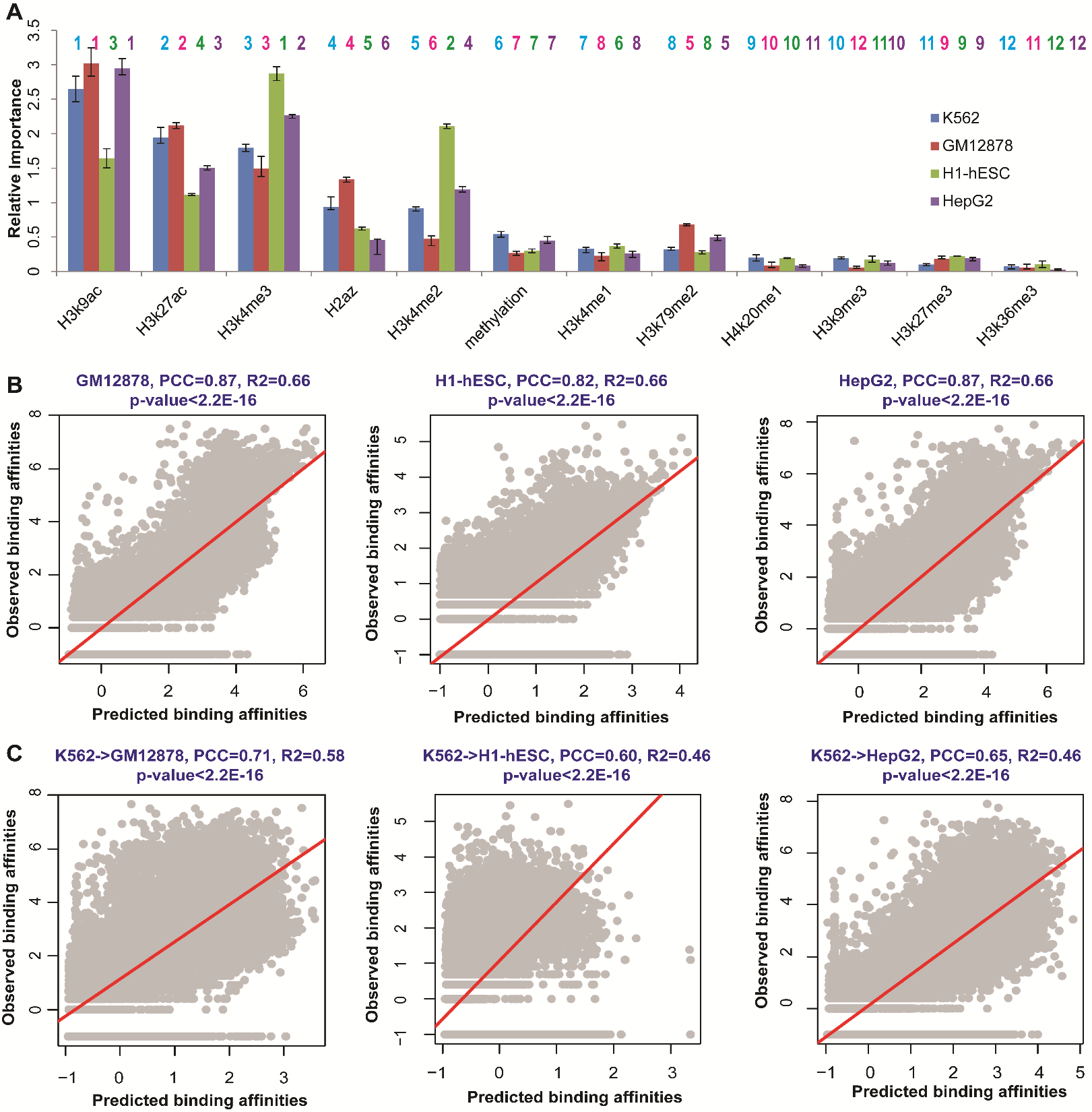


**Supplemental Figure S9:** Epigenetic modification models for predicting TF binding affinity in each gene location are cell line-specific. (A) Relative importance of epigenetic modifications in predicting binding affinities of YY1 in the four (blue: K562, red: GM12878, green: H1-hESC, and purple: HepG2) cell lines. The orders of relative importance of epigenetic features to model TF binding affinity across cell lines were shown on the top of each bar with corresponding colors. (B) Comparisons between predicted and observed binding affinities for YY1 within upstream -100 to -1bp region (Bin -1) in the GM12878, H1-hESC and HepG2 cell lines. The RF model was trained and applied in the same cell line. (C) The epigenetic modification models learned from the K562 cell line were applied to other cell lines (GM12878, H1-hESC, HeLa-S3 and HepG2) for predictions of YY1 binding affinities in upstream -100 to -1bp region (Bin -1). The epigenetic modification levels at the same genome location were used as inputs. The decreased prediction accuracies, compared to the predictions with models obtained from the same cell line, indicates that the epigenetic modification models for predicting TF binding affinities are cell line-specific.


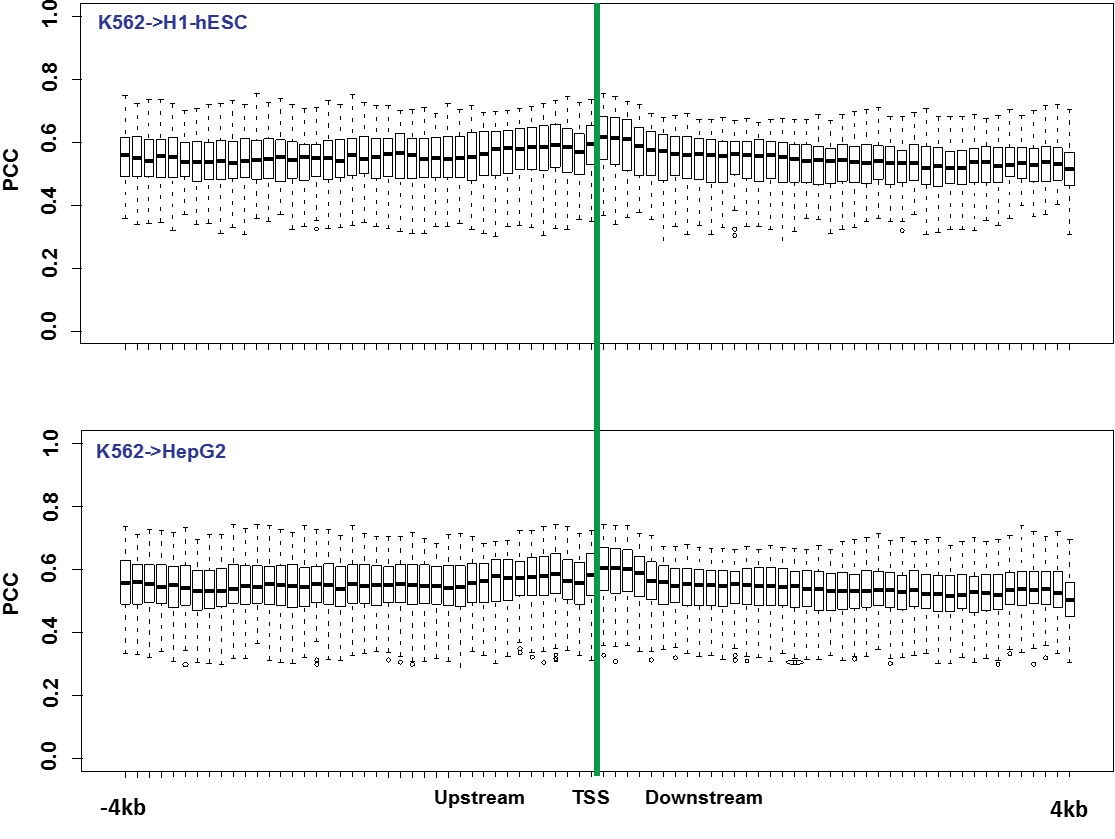


**Supplemental Figure S10:** Box plots for prediction accuracies of TF binding affinities with epigenetic modification models obtained from the K562 cell line and applied to the H1-hESC and HepG2 cell lines. Various numbers of TFs are included in each figure based on the availability of ChIP-Seq data (Supplemental Table S1). In all 80 bins, prediction accuracies are dramatically decreased, compared to the predictions with models obtained in the same cell line, indicating that epigenetic modification models for TF binding predictions are cell line-specific.


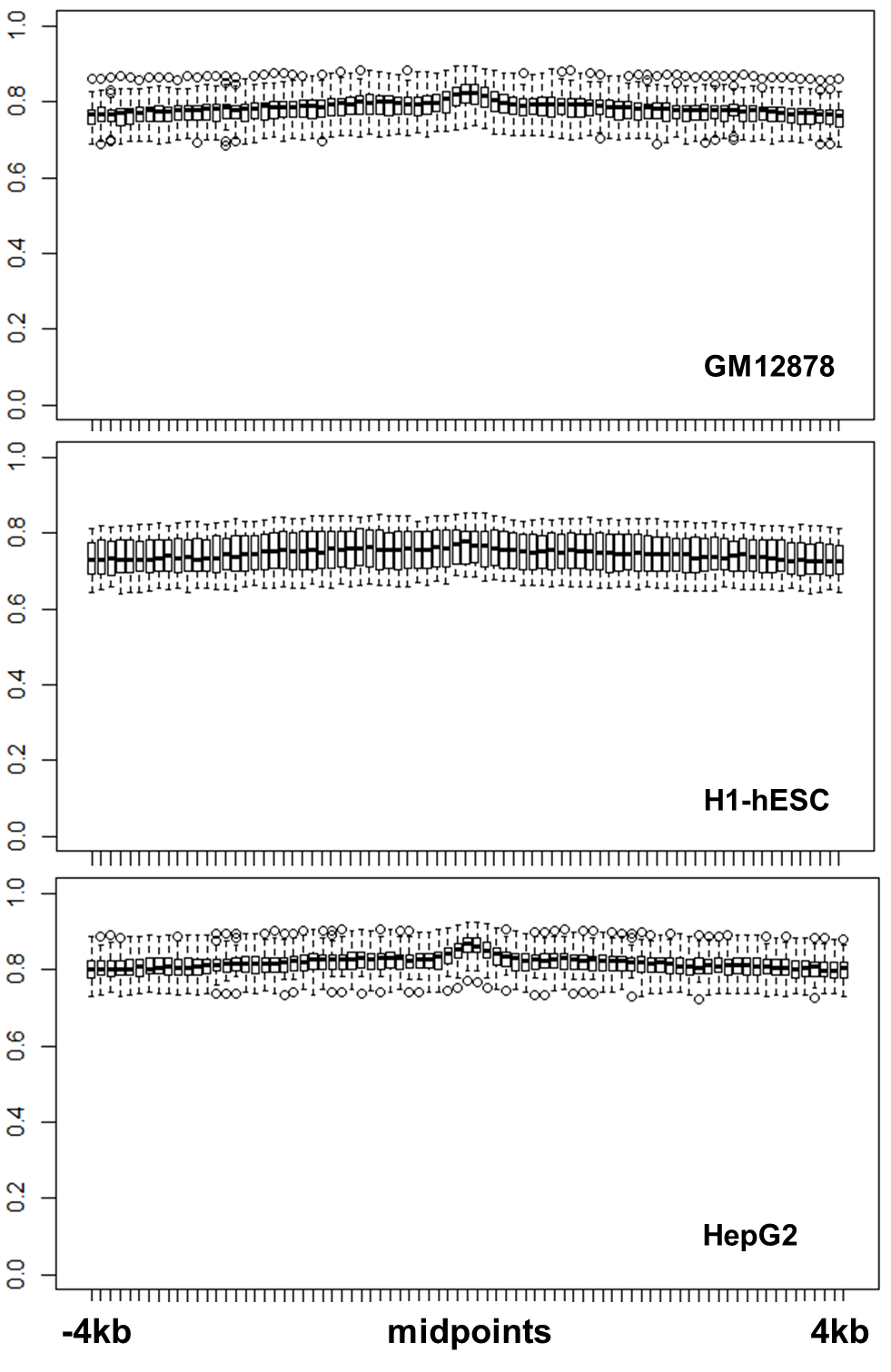


**Supplemental Figure S11:** Box plots for the prediction accuracies of TF binding affinities with epigenetic modifications across 8k-bp regions of gene enhancers in the GM12878, H1-hESC, and HepG2 cell lines. The RF model was employed in each bin for each TF and cell line. Various numbers of TFs were included in each figure due to the availability of ChIP-Seq data (Supplemental Table S1). High accuracies indicate that TF binding affinities can be precisely reflected by epigenetic modifications in any cell condition.


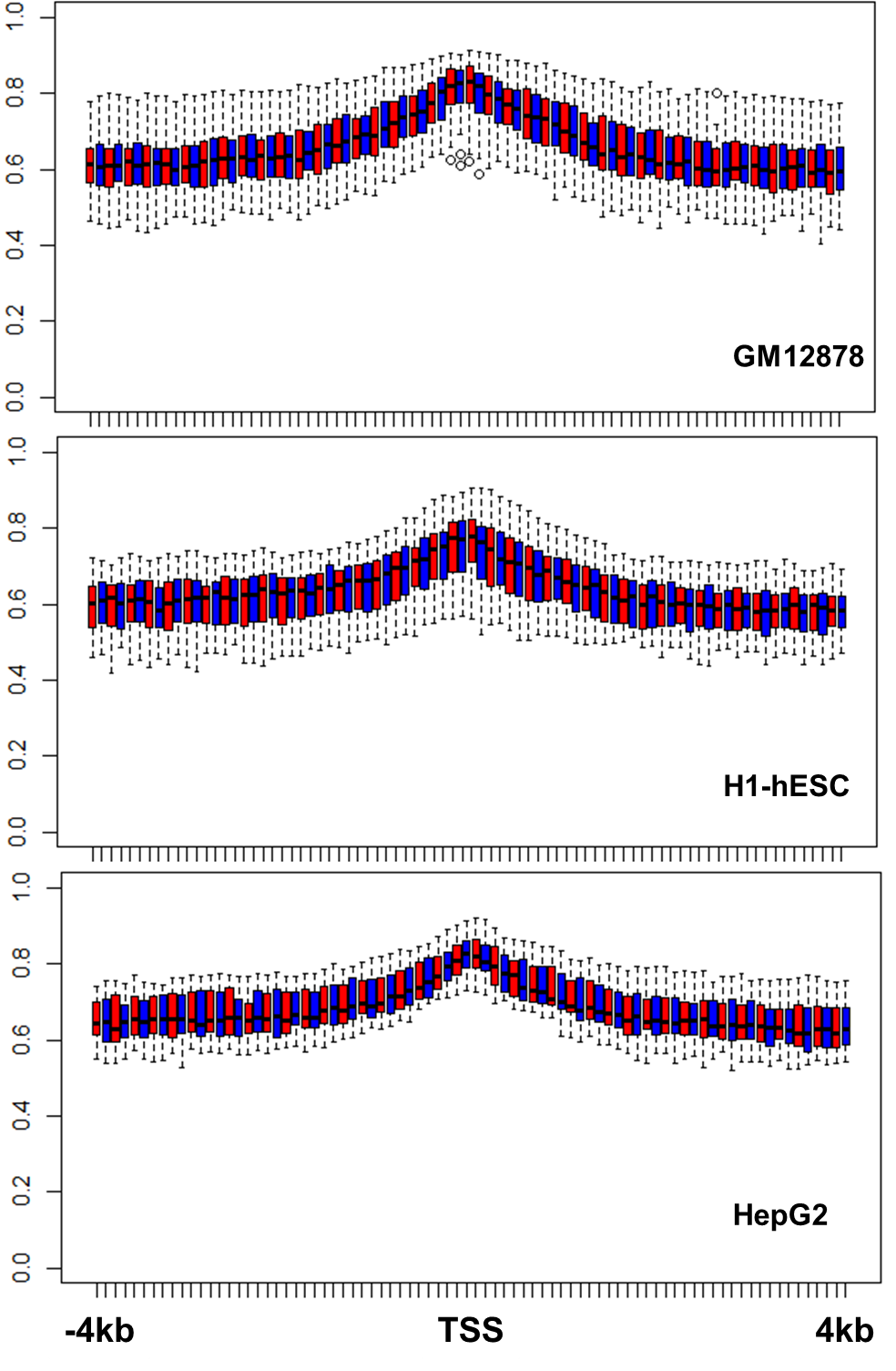


**Supplemental Figure S12:** Box plots for the prediction accuracies of TF binding affinities with epigenetic modification models obtained from protein-coding genes and applied to non-coding genes in the GM12878, H1-hESC and HepG2 cell lines. Red: epigenetic modification models were trained and applied to non-coding genes; Blue: epigenetic modification models were trained for protein-coding genes and applied to non-coding genes.


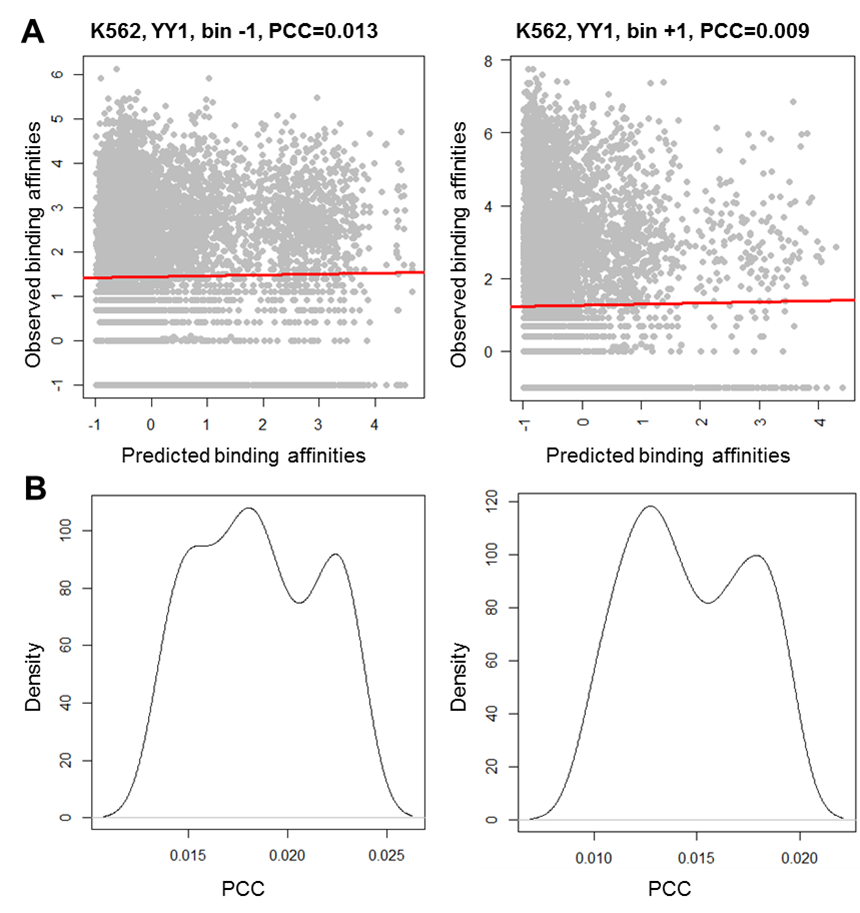


**Supplemental Figure S13**: Prediction accuracies of MRL models for YY1 using the histone modification and DNA methylation levels in upstream -100 to -1bp (Bin -1) and downstream 1 to 100bp regions (Bin +1). Epigenetic modification signals and TF binding affinities were shuffled based on gene annotations. (B) Distribution of prediction accuracies when the shuffling process was repeated 50 times.


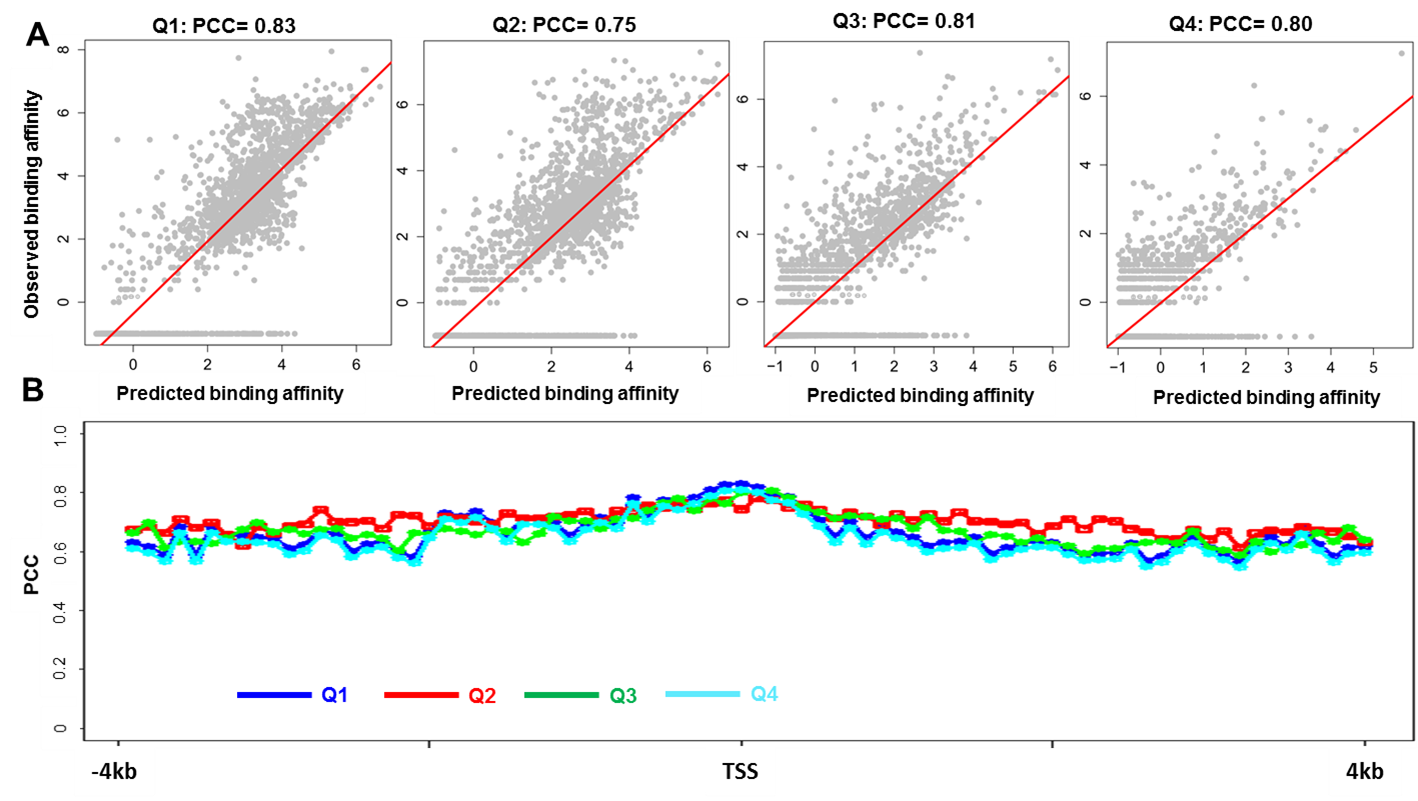


**Supplemental Figure S14**: Comparisons of prediction accuracies across different groups of genes based on their expression levels. (A) Comparisons for YY1 binding affinities in upstream -100 to -1bp (Bin -1) region. Genes were separated into 4 quartiles based on expression levels. (B) Prediction accuracies (PCCs) across 80 bins. Blue: Q1, Red: Q2, Green: Q3 and Cyan: Q4.


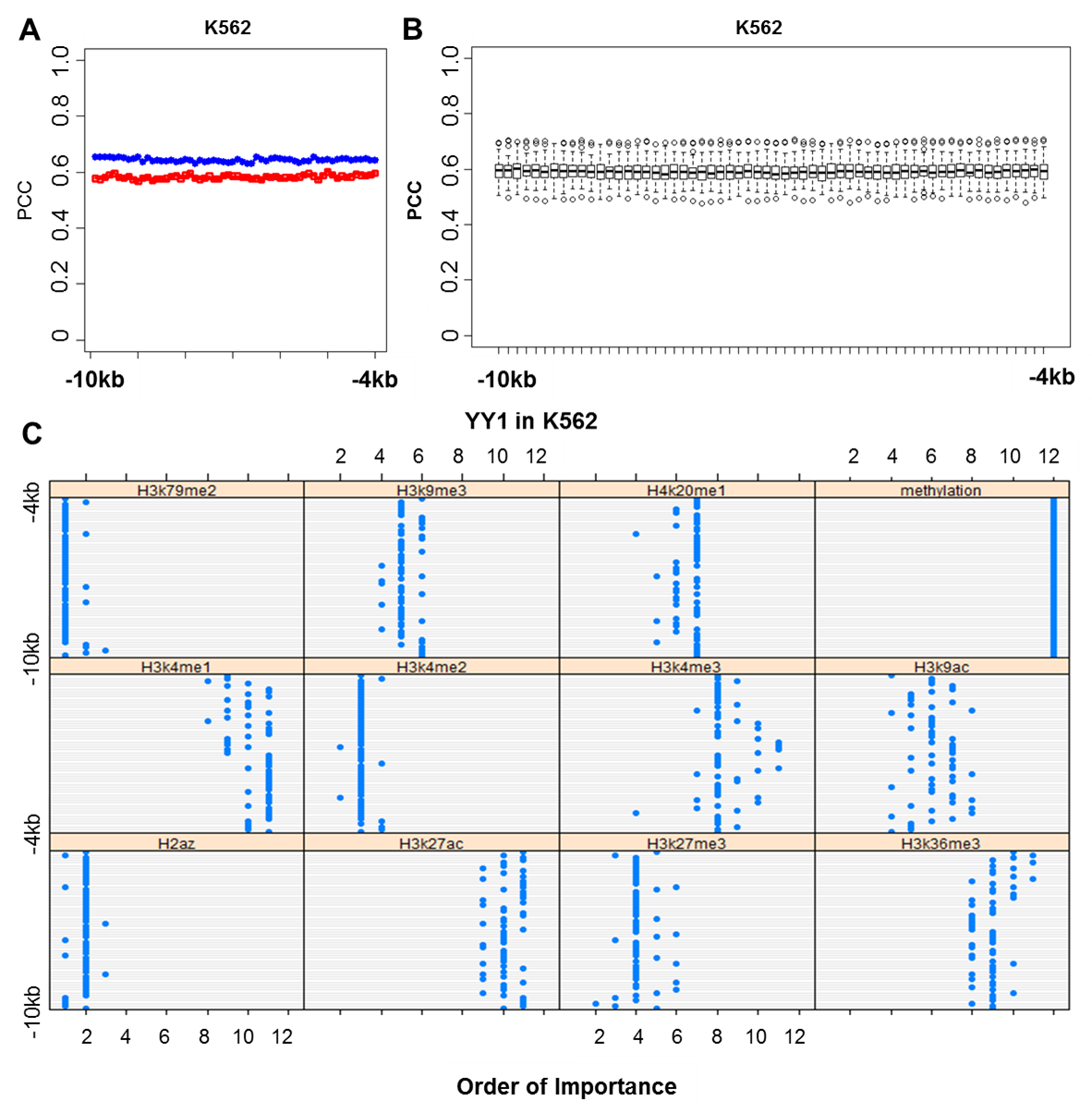


**Supplemental Figure S15**: Performance of epigenetic modification models in upstream 10k and 4k-bp region. Prediction accuracies for (A) YY1 (blue) and ATF3 (red), and (B) all TFs in K562 cell lines. (C) Relative importance of epigenetic modifications in predicting YY1 bindings in K562 cell line.


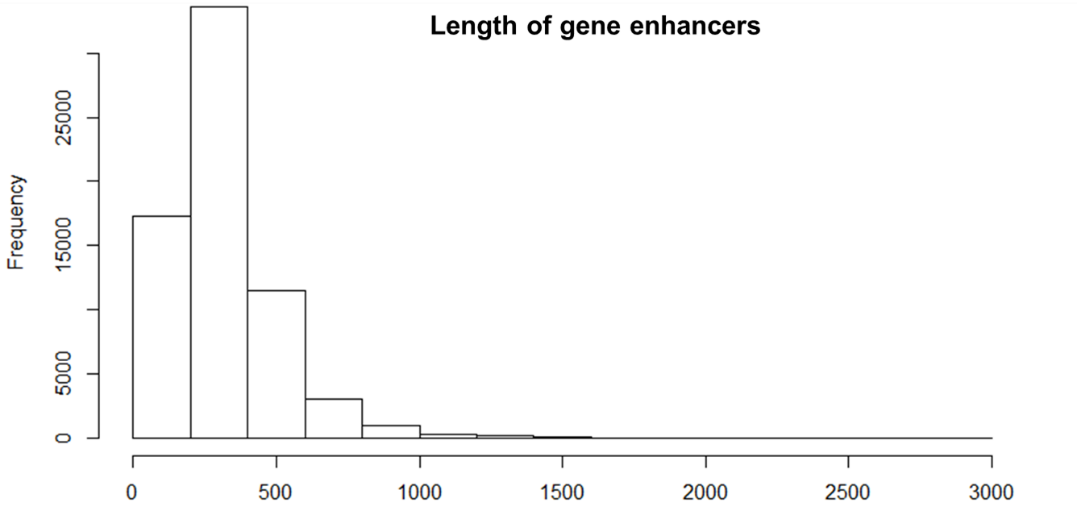


**Supplemental Figure S16**: Lengths of gene enhancers. Enhancer information was obtained from the FANTOM5 consortium ([4](#_ENREF_4)).


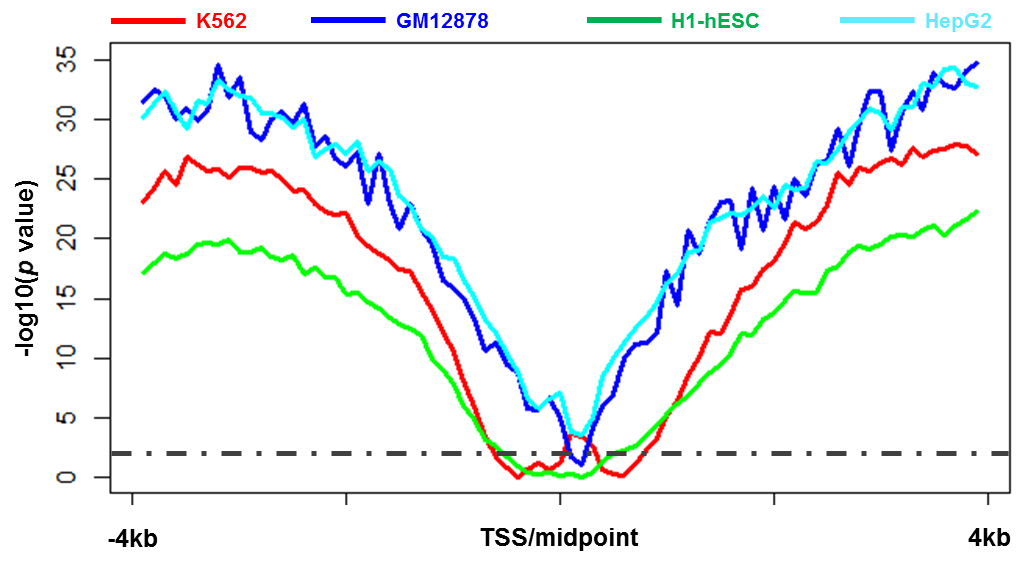


**Supplemental Figure S17**: Comparison of prediction accuracies of TF binding affinities between promoters and enhancers. Two-tailed Student’s *t* test was applied to the prediction accuracies in each bin and each cell line, and –log10 transformation was performed to *p* value. Predictions are generally better in enhancers (Figures 4D, 5C, Supplemental Figures S3 and S12), especially in the regions far from center (*p* values < 0.05, marked with the black dotted line).


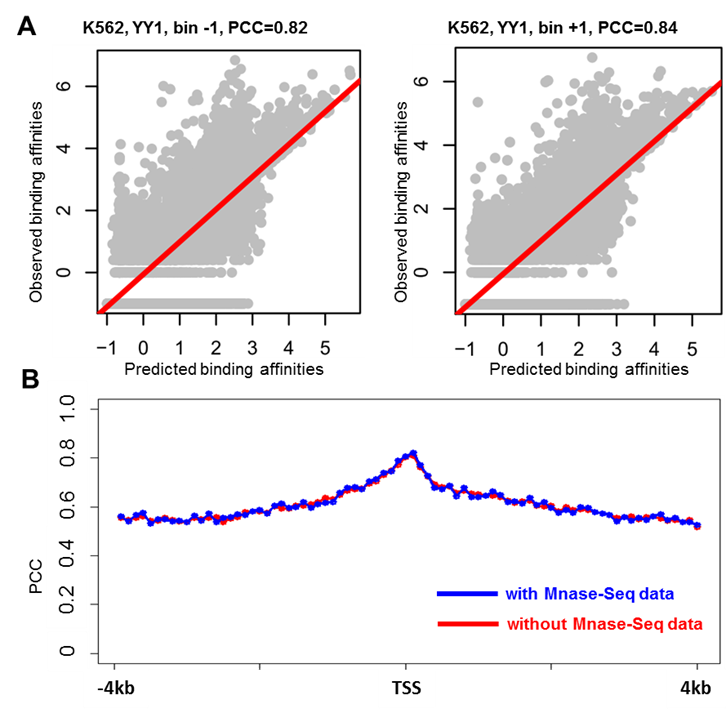


**Supplemental Figure S18**: Comparisons between predicted TF binding affinities and experimental measurements from the ChIP-Seq data. The MRL models were employed to predict the binding affinities of YY1 using histone modification, DNA methylation levels and nucleosome positioning signals. (A) Prediction accuracies in upstream -100 to -1bp (Bin -1) and downstream 1 to 100bp regions (Bin +1). (B) Comparison of prediction accuracies when using (Blue curve) or not using the nucleosome positioning signals (Red curve) across 80 bins centered at TSSs.

*
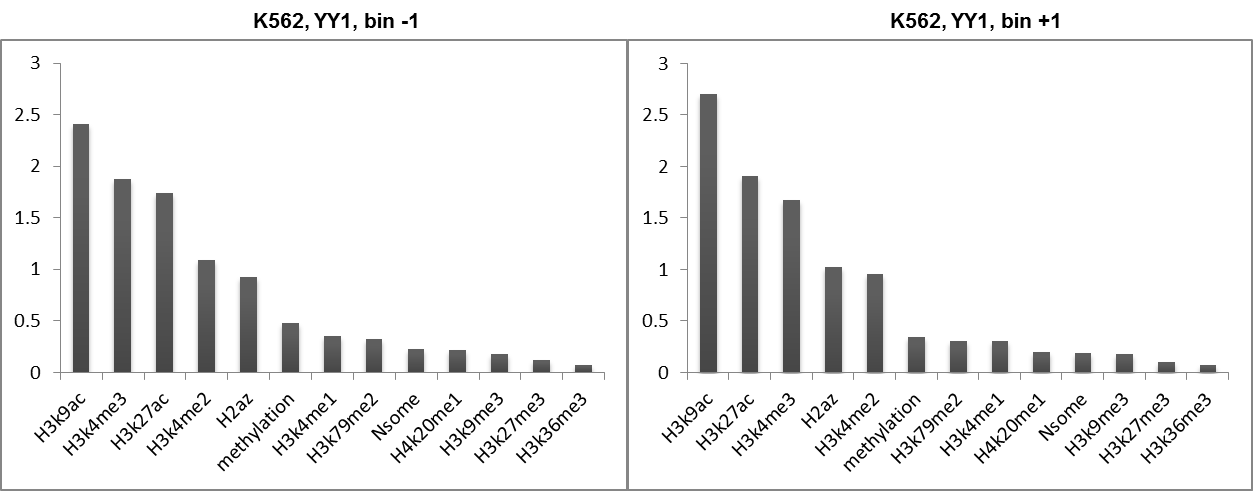
*

**Supplemental Figure S19**: Relative importance of each feature including nucleosome occupancy for predicting YY1 binding affinities (in K562 cell line) in -100 to -1bp (Bin -1) and 1 to 100bp (Bin +1) regions.

**3. Supplemental Table S1**

**Supplementary Table S1:** List of TFs included in the analyses, including 75, 69, 42, and 41 TFs for the K562, GM12878, H1-hESC and HepG2 cell lines. The TFs existing in both K562 and one of other three cell lines were highlighted, including 34, 27, and 24 TFs in the GM12878, H1-hESC, and HepG2 cell lines, respectively.

| **K562** | **GM12878** | **H1-hESC** | **HepG2** | **K562** | **GM12878** | **H1-hESC** | **HepG2** |
| --- | --- | --- | --- | --- | --- | --- | --- |
| ATF3 | ATF2 | ATF3 | ATF3 | P300 | P300 | TEAD4 | YY1 |
| BCL3 | ATF3 | BCL11A | BHLHE40 | PCAF | PAX5 | USF-1 | ZBTB33 |
| BCLAF1 | BATF | BRCA1 | BRCA1 | PHF8 | PBX3 | USF2 | ZBTB7A |
| BRG1 | BCL11A | CHD1 | CEBPB | PLU1 | PML | YY1 |  |
| CBP | BCL3 | CHD2 | CEBPD | PML | POL2 |  |  |
| CBX2 | BCLAF1 | CHD7 | CHD2 | POL2 | POL3 |  |  |
| CBX3 | BHLHE40 | CTCF | CREB1 | PU.1 | POU2F2 |  |  |
| CDP | CDP | E2F6 | CTCF | RAD21 | PU.1 |  |  |
| CEBPB | CEBPB | EGR-1 | ELF1 | REST | RAD21 |  |  |
| CEBPD | CHD1 | EZH2 | EZH2 | RFX5 | RFX5 |  |  |
| CTCF | CHD2 | FOSL1 | FOSL2 | RNF2 | RUNX3 |  |  |
| CTCFL | COREST | GABP | FOXA1 | SAP30 | RXRA |  |  |
| E2F4 | CREB1 | GTF2F1 | FOXA2 | SETDB1 | SIN3A |  |  |
| E2F6 | CTCF | JMJD2A | GABP | SIN3AK-20 | SIX5 |  |  |
| EGR-1 | E2F4 | JUND | HDAC2 | SIRT6 | SMC3 |  |  |
| ELF1 | EBF1 | NANOG | HEY1 | SIX5 | SP1 |  |  |
| ETS1 | EGR-1 | NRF1 | HNF4A | SP1 | SREBP1 |  |  |
| FOSL1 | ELF1 | NRSF | HNF4G | SP2 | SREBP2 |  |  |
| GABP | ELK1 | P300 | IRF3 | SRF | SRF |  |  |
| GATA1 | ETS1 | PHF8 | JUND | STAT5A | STAT1 |  |  |
| GATA2 | EZH2 | PLU1 | MAFF | SUZ12 | STAT3 |  |  |
| GTF2F1 | FOXM1 | POL2 | MAX | TAF1 | TAF1 |  |  |
| HDAC1 | GABP | POU5F1 | MXI1 | TAF7 | TBLR1 |  |  |
| HDAC2 | IRF3 | RAD21 | MYBL2 | TBLR1 | TBP |  |  |
| HEY1 | IRF4 | RFX5 | NFIC | TBP | TCF12 |  |  |
| JUND | JUND | RXRA | NR2F2 | THAP1 | TCF3 |  |  |
| KAP1 | MAFK | SAP30 | NRSF | TR4 | USF-1 |  |  |
| LSD1 | MAX | SIN3A | P300 | TRIM28 | USF2 |  |  |
| MAFK | MAZ | SIRT6 | POL2 | UBF | WHIP |  |  |
| MAX | MEF2A | SIX5 | RAD21 | USF-1 | YY1 |  |  |
| MAZ | MEF2C | SP1 | RXRA | YY1 | ZBTB33 |  |  |
| MEF2A | MTA3 | SP4 | SP1 | ZBTB33 |  |  |  |
| MXI1 | MXI1 | SRF | SP2 | ZBTB7A |  |  |  |
| NF-YB | NFATC1 | SUZ12 | SRF | ZNF143 |  |  |  |
| NR2F2 | NF-E2 | TAF1 | TAF1 | ZNF274 |  |  |  |
| NRF1 | NFIC | TAF7 | TCF12 | ZNF384 |  |  |  |
| NRSF | NRF1 | TBP | TEAD4 | ZNF-MIZD-CP1 |  |  |  |
| NSD2 | NRSF | TCF12 | USF-1 |  |  |  |  |

**REFERENCE**

1. Teytelman, L., Thurtle, D.M., Rine, J. and van Oudenaarden, A. (2013) Highly expressed loci are vulnerable to misleading ChIP localization of multiple unrelated proteins. *Proc Natl Acad Sci U S A*, **110**, 18602-18607.

2. Park, D., Lee, Y., Bhupindersingh, G. and Iyer, V.R. (2013) Widespread misinterpretable ChIP-seq bias in yeast. *PLoS One*, **8**, e83506.

3. Kundaje, A., Kyriazopoulou-Panagiotopoulou, S., Libbrecht, M., Smith, C.L., Raha, D., Winters, E.E., Johnson, S.M., Snyder, M., Batzoglou, S. and Sidow, A. (2012) Ubiquitous heterogeneity and asymmetry of the chromatin environment at regulatory elements. *Genome Res*, **22**, 1735-1747.

4. Andersson, R., Gebhard, C., Miguel-Escalada, I., Hoof, I., Bornholdt, J., Boyd, M., Chen, Y., Zhao, X., Schmidl, C., Suzuki, T. *et al.* (2014) An atlas of active enhancers across human cell types and tissues. *Nature*, **507**, 455-461.
